# Cholesterol Derivatives Regulate Adenylyl Cyclase 7 Activity by Binding CARC and CRAC Motifs in the Cytosolic Subunits

**DOI:** 10.1021/acsomega.5c00741

**Published:** 2025-08-14

**Authors:** Radim Jaroušek, Petra Dad’ová, Alexandra Litvinchuk, Leticia Dobler, Lukáš Kubala

**Affiliations:** 1 Department of Experimental Biology, Faculty of Science, 37748 Masaryk University, Kotlářská 2, 602 00 Brno, Czech Republic; 2 Department of Biophysics of the Immune System, 86853 Institute of Biophysics, Czech Academy of Sciences, Královopolská 135, 612 00 Brno, Czech Republic; 3 International Clinical Research Center, Center of Biomolecular and Cellular Engineering, St. Anne’s University Hospital Brno, Pekařská 53, 656 91 Brno, Czech Republic

## Abstract

Transmembrane adenylyl cyclases (tmACs; ACs) are enzymes that synthesize cyclic AMP (cAMP), which is a key molecule in cellular signaling. Disruptions in AC activity can lead to long-term shifts in cAMP levels associated with various pathologies. In our study, we analyzed AC primary sequences and identified cholesterol-binding CARC and CRAC motifs located in conserved cytosolic regions, a surprising finding for motifs that are typically membrane-associated. Focusing on AC7, we mapped these motifs within its predicted structure and performed docking studies with cholesterol derivatives (hydrocortisone, dexamethasone, and 25-hydroxycholesterol). Our results showed that these molecules predominantly bind to the forskolin (FSK) binding site, which contains two CARC motifs. Using membranes overexpressing AC7, we observed that all three derivatives significantly decreased FSK-mediated AC7 activity by up to 55%. This suggests that cholesterol derivatives might interact with CARC and CRAC motifs to regulate AC7 function and underscore the potential of cholesterol derivatives as natural modulators as well as provide a compelling basis for future exploration of cholesterol derivatives as possible therapeutic regulators of AC7.

## Graphical abstract

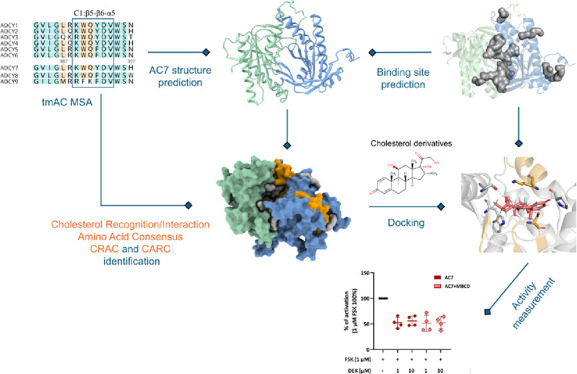

## Introduction

Cyclic adenosine monophosphate (cAMP) is a secondary messenger that participates in cell signaling and is involved in various physiological and pathophysiological processes. The most widely known processes include embryogenesis, cardiac function, pain reception, learning, and aging.^1–3^ The synthesis of cAMP in the cell is catalyzed by transmembrane adenylyl cyclase enzymes (tmACs; ACs). Human ACs are generally divided into families according to the homology of primary sequences and regulatory properties.^1,3^ AC1, AC3, and AC8 belong to the type 1 group.^1,4^ AC2, AC4, and AC7 belong to the type 2 group, and AC5 and AC6 belong to the type 3 group.^1,2,5^ Although there is high sequence homology between the primary sequences of AC catalytic domains, many different residues and motifs are group specific. In all ACs, cytosolic domains are highly structurally homologous and form a heterodimeric structure responsible for catalytic activity.^5–7^ Generally, all ACs are activated *in vivo* by the Gαs subunit of G-proteins.^1,5,8^ AC2, AC4, and AC7 (type 2 group) are only conditionally activated by Gβγ subunits, and AC7 is the only isoform that could be activated by protein kinase C (PKC).^2,3,9^ Interestingly a small molecule of forskolin (FSK) from the plant *Coleus Forskolii* has been used for many years as a direct activator/regulator of ACs. FSK is known to bind a highly conserved site, mimicking an activation mechanism via Gαs.^10,11^

Numerous clinically approved drugs function through G-protein-coupled receptors (GPCRs), which modulate AC activity or target enzymes involved in cAMP degradation. Despite the extensive characterization of these pathways, an endogenous molecule functionally analogous to FSK remains unidentified. Discovering this molecule could significantly advance our understanding of the regulatory mechanisms underlying AC dysfunction, a feature common to multiple diseases. Among the AC isoforms, AC7 has emerged as a particularly attractive therapeutic target due to its specific expression in brain and immune cells, linking it directly to conditions such as alcoholism, depression, and autoimmune disorders.^1–3,12^ Further highlighting its biological importance, AC7 deficiency uniquely results in embryonic lethality in knockout mouse models.^13^ Targeting AC7 thus represents a promising therapeutic strategy for managing diseases associated with aberrant cAMP signaling.

Initially, the cholesterol molecule was thought to cause only nonspecific effects on membranes, such as the changes of fluidity, permeability, or formation of microdomains.^14^ However, later it was demonstrated that this lipid could specifically interact with transmembrane proteins and affect both their structure and function.^15^ Cholesterol binding occurs through two well-defined motifs: the CRAC (Cholesterol Recognition/Interaction Amino Acid Consensus) motif, characterized by the sequence L/V–X1–5–Y–X1–5–K/R,^16^ and the CARC motif, a mirrored version identified as K/R–X1–5–Y/F–X1–5–L/V.^17^ Given the significance of these motifs, we investigated their presence in AC sequences and their potential influence on the functional activity of AC7.

Considering the absence of an endogenous equivalent to the allosteric regulator FSK, identifying a native regulatory molecule became particularly relevant. Although transmembrane regions of AC isoforms generally exhibit low sequence homology, the presence of CARC and CRAC motifs within cytosolic domains and the FSK binding site offers a basis for investigating the role of these motifs in AC regulation. In our study, we conducted comprehensive *in silico* analyses, including multiple sequence alignment (MSA), binding site prediction, *AlphaFold2* structure modeling, and molecular docking, to explore if CARC and CRAC motifs may play a role in the AC7 regulation. Our results demonstrate that regions comprising these motifs are conserved among various AC isoforms and are critical for AC catalytic activity and regulation. These computational findings were further investigated experimentally by using cell membranes overexpressing the AC7 isoform. To further assess the regulatory potential of the CARC and CRAC motifs, we tested cholesterol-related compounds and measured AC7 activity following treatment with three cholesterol derivatives: hydrocortisone, dexamethasone, and 25-hydroxycholesterol. These experiments were designed to elucidate their impact on enzymatic function and provide insight into the potential ligand-mediated modulation of AC7.

## Methods and Experimental Procedures

### Multiple Sequence Alignment

MSA was built in R using the MSA package^18^ using validated human protein sequences of AC1-AC9 from *UniprotKB* (Table 1). Created MSA was imported into *Geneious* software^19^ (24.1.13) and enriched by annotations from the *PROSITE*, *UniprotKB*, and *Pfam* databases [Bibr ref20]−[Bibr ref21] [Bibr ref22] (2024). For secondary structure prediction, the *EMBOSS* ^23^ tool plugin was used. Annotations with 80% or more sequence similarity were unified and renamed. Renamed annotations: ATP as substrate binding, NBS for the nucleotide binding site, and DIMER for interacting residues (interface) between C1 and C2 cytosolic subunits. M1/M2 describes residues participating in the ion binding. The residue colors in the MSA correspond to the identity between the AC1–AC9 sequences. The final figures were rearranged in *GIMP* (2.1). software.^24^

**Table 1:** Adenylate Cyclase Sequences Used for the Construction of MSA

isoform	*UniprotKB* identifier	*UniprotKB* name	protein name
AC1	Q08828	ADCY1_HUMAN	adenylate cyclase type 1
AC2	Q08462	ADCY2_HUMAN	adenylate cyclase type 2
AC3	O60266	ADCY3_HUMAN	adenylate cyclase type 3
AC4	Q8NFM4	ADCY4_HUMAN	adenylate cyclase type 4
AC5	O95622	ADCY5_HUMAN	adenylate cyclase type 5
AC6	O43306	ADCY6_HUMAN	adenylate cyclase type 6
AC7	P51828	ADCY7_HUMAN	adenylate cyclase type 7
AC8	P40145	ADCY8_HUMAN	adenylate cyclase type 8
AC9	O60503	ADCY9_HUMAN	adenylate cyclase type 9

### *AlphaFold2* Structure Prediction

The structure of human AC7 catalytic subunits was predicted using *AlphaFold2* software^25^ accessed through *ColabFold* ^26^ (1.5) with default parameters (mmseqs2-uniref; unpaired–paired) and compared to the existing PDB hybrid structures of 3c16 ^27^ and 1azs^7^ using *Pymol* (2.3.5) software.^28^ The whole sequence used for the structure prediction can be found in Table S1. The predicted AC7 model includes secondary structure elements, which were described in figures and compared to the 3c16 experimental structure for validation. The list of all secondary structure elements and their positions is shown in Table S2.

The optimized model of AC7 was used for the binding site prediction via *DeepSite* ^29^ with default parameters. All structures for *in silico* analysis were visualized in *Pymol* (2.3.5) software^28^ and *Flare* ^30^ (10.0.0). Created ray images with transparent background in 2000dpi were rearranged in *GIMP* (2.1) software.^24^

### Docking of Molecules

Molecules of cholesterol and tested derivatives for *silico* analysis were created in *Avogadro* (1.1.0) software^31^ based on validated structures from *PubChem* and stabilized by the MFF94 force field. All hydrogens and covalently bound ligands were removed from the structure before docking. Afterward, the *AlphaFold2* structure was optimized and cholesterol molecules (protonated) docked using *DockThor* software^32^ with default parameters (1mil evaluations; size population—750; 24 runs).

For blind docking of cholesterol derivatives, *DiffDock* (1.1.2) software was used.^33^ Final structures were also visualized by *Pymol* (2.3.5) software in combination with *ChimeraX* (1.10)^34^ and *Flare* (10.0.0).^30^ The raw data from *in silico* analysis including running parameters are available through: https://github.com/RadJarous/AC7_regulation or 10.5281/zenodo.14540567.

### Residue Interaction Plot

A PDB files with bound ligand molecules were imported into *Flare* (10.0.0).^30^ For the purpose of the binding description, both the ligand and receptor (AC7 model) were aggregated. The 2D plot was exported to a *svg* file, rescaled, and adjusted using *GIMP* (2.1) software.^24^

### Cell Cultivation

Human embryonic kidney cells (HEK293) (ATCC, USA) were cultured in Dulbecco’s modified Eagle’s medium (DMEM) (Thermo Fisher Scientific, USA) containing 10% fetal calf serum (PAA Laboratories, Austria), 10,000 IU/mL penicillin, and 1000 μg/mL streptomycin (all from PAA Laboratories, Austria) to 80% confluence. Cells were maintained at 37 °C in 5% CO_2_, and the culture medium was changed every 3 days.

### Cell Transfection for AC

HEK293 cells were transfected with pCMV6-Entry (Origene, USA), which encoded the AC7 sequence with a DDK sequence. After 48 h, the medium was exchanged, and the cells were treated with a selection antibiotic G418 (2.2 mg/mL) for a period of 3–4 weeks to confirm stable transfection. Selected clones were further cultured and used for other experiments. HEK 293 cell cultures without a plasmid were used as a control. Cell clones were checked by Western blotting with antibody against the DDK sequence.

### Protein Assay

Total protein quantification was finally determined by a Thermo Fisher Scientific Pierce Detergent Compatible Bradford Assay Kit (Thermo Fisher Scientific, USA). The protein assay was performed in a microplate format, and absorbance values for standard and membrane samples were measured at 595 nm using a spectrophotometer (Sunrise, Tecan, Switzerland).

### SDS-PAGE and Western Blotting Assay

Protein expression analysis of the DDK flag tag was performed as described here. Membrane samples were lysed using lysis buffer (8 M urea, 2 M thiourea, 0.05 M Tris, 3% SDS, DTT 75 mM, 0.004% bromophenol blue, pH 6.8) to set the protein concentrations by 1 mg/mL. SDS-PAGE gel running (7.5%) was performed for 10–15 μg of total protein samples. Proteins were transferred onto a poly­(vinyl difluoride) membrane (PVDF, Merck Millipore; Darmstadt, Germany). PVDF membranes were blocked by 5% bovine serum albumin (in TBS-T buffer (Tris, 0.05% Tween20). The detection of overexpressed proteins was performed using the primary anti-DDK antibody (TA50011-1, Origene, USA) and antimouse HRP-conjugated as a secondary antibody (Cell Signaling, Danvers, MA, USA; 7076S). Received densities were quantified by scanning densitometry and expressed in arbitrary units determined (obtained) by *ImageJ* software (NIH, USA).

### Cell Membrane Isolation

For membrane isolation, usually six 150 mm Petri dishes with confluent HEK 293 cells with overexpressed AC7 were prepared. After the medium was removed and cells were washed with PBS, dishes with cells were frozen at −80 °C. The next day, cells were thawed, and ice-cold lysis buffer (20 mM HEPES, 1 mM EDTA, 2 mM MgCl_2_.6H_2_0, 1 mM DTT, 250 mM sucrose) and a protease inhibitor cocktail were added. The lysis of cells was supported by homogenization in a Dounce homogenizer (Sigma-Aldrich, St. Louis, MO, USA), and the subsequent suspension was centrifuged at 1800*g* for 5 min at 4 °C for nuclei sedimentation. The supernatant was transferred into ultracentrifuge tubes. The ultracentrifugation step was performed in an Optima LE-80K Preparative Ultracentrifuge (Beckman Coulter, Brea, CA, USA) at 23,000 rpm for 20 min at 4 °C using a SWTi55 rotor. Subsequently, the protein concentration was determined by the Bradford assay using the Compatible Bradford Assay Kit (Thermo Fisher Scientific, Waltham, MA, USA). Then, the aliquots of the membranes were frozen and stored at −80 °C.

### Determination of AC Activity

AC activity was determined based on quantitative measurement of produced cAMP using Time-Resolved Fluorescence Resonance Energy Transfer (TR-FRET) (Lance Ultra cAMP kit from PerkinElmer, USA). The assay is based on competition between the europium (Eu) chelate-labeled cAMP tracer and the cAMP sample for binding sites on cAMP-specific monoclonal antibodies. Prepared membrane fractions were diluted with lysis buffer (1 mg/mL) (see membrane preparation), followed by dilution in stimulation buffer to a final concentration per well. The stimulation buffer consisted of Hank’s balanced salt solution (Life Technologies, USA), 5 mM HEPES, 0.5 mM IBMX, 0.1 mM ATP, 10 mM MgCl_2_.6H_2_0, and 0.1% stabilized BSA (supplied by the kit manufacturer). The concentrations of Ca/Mg ions and ATP were optimized. A 384-well black plate format was used (Nunc, Thermo Fisher Scientific, USA).

FSK was diluted in the stimulation buffer to final concentrations ranging from 120 to 0.012 μM and incubated with membranes in wells at room temperature for 30 min. The reaction was stopped by adding the fluorescence probes Europium-tracer and U-Light at concentrations recommended in the kit. The probes were incubated together at room temperature for 1 h in darkness. The fluorescence signal, which is inversely proportional to the overall amount of cAMP, was read immediately using a Spark M10 spectrophotometer (Tecan, Switzerland) with excitation fluorescence at 320 nm and emission fluorescence at 620 and 665 nm. The lag time was 150 μs, and the integration time was 500 μs. The efficacy for each compound (derivative) was determined by dividing the stimulation obtained for a distinct concentration of the derivative by the maximum stimulation obtained by treatment with 120 μM FSK (100%) expressed in percent.

### Cholesterol Depletion and Its Membrane Content Quantification

Cholesterol depletion was done in HEK 293 cells with methyl-β-cyclodextrin (MBCD, Sigma-Aldrich, St. Louis, MO, USA) at a ratio of 1:10. Isolation membranes of AC7 HEK293 were incubated with the medium containing 5 mM MBCD for 1 h at 37 °C. Cholesterol content was determined by fluorometry using the Amplex Red Cholesterol Assay quantitation kit (Thermo Fisher Scientific, Oslo, Norway) following the manufacturer’s instructions, and the product was measured in an infinite microplate reader (Tecan, Männedorf, Switzerland) with a 530 nm/590 nm filters.

### Statistics

Data are presented as the mean ± standard error of the mean (SEM). Statistical differences between mean values were determined by using GraphPad Prism-9 software (GraphPad software, La Jolla, CA, USA). A one-sample *t* test was used to compare values expressed as percentages. In the case of the one-sample *t* test, the number of independent repeats (*n*) is given in each figure legend.

## Results

### Cholesterol-Binding Motifs Are Located in Catalytic Subunits of AC7

Our analysis showed that human AC isoforms contain multiple CARC and CRAC motifs within their cytosolic domains. All identified motifs and their sequence positions are summarized in Table 2. To better understand their structural localization, we used the predicted 3D structure of AC7 catalytic subunits. The analysis revealed that most of the CARC and CRAC motifs are located in highly conserved regions of the C1 and C2 subunits. The localization of all identified motifs is shown in Figure 1 and Figure S1. Specifically, two CARC motifs were identified within the C1 cytosolic domain (amino acid region 197-594), while the remaining CARC and CRAC motifs were found in the C2 cytosolic domain (amino acid region 815-1080) (Table 2).

**Figure 1:**
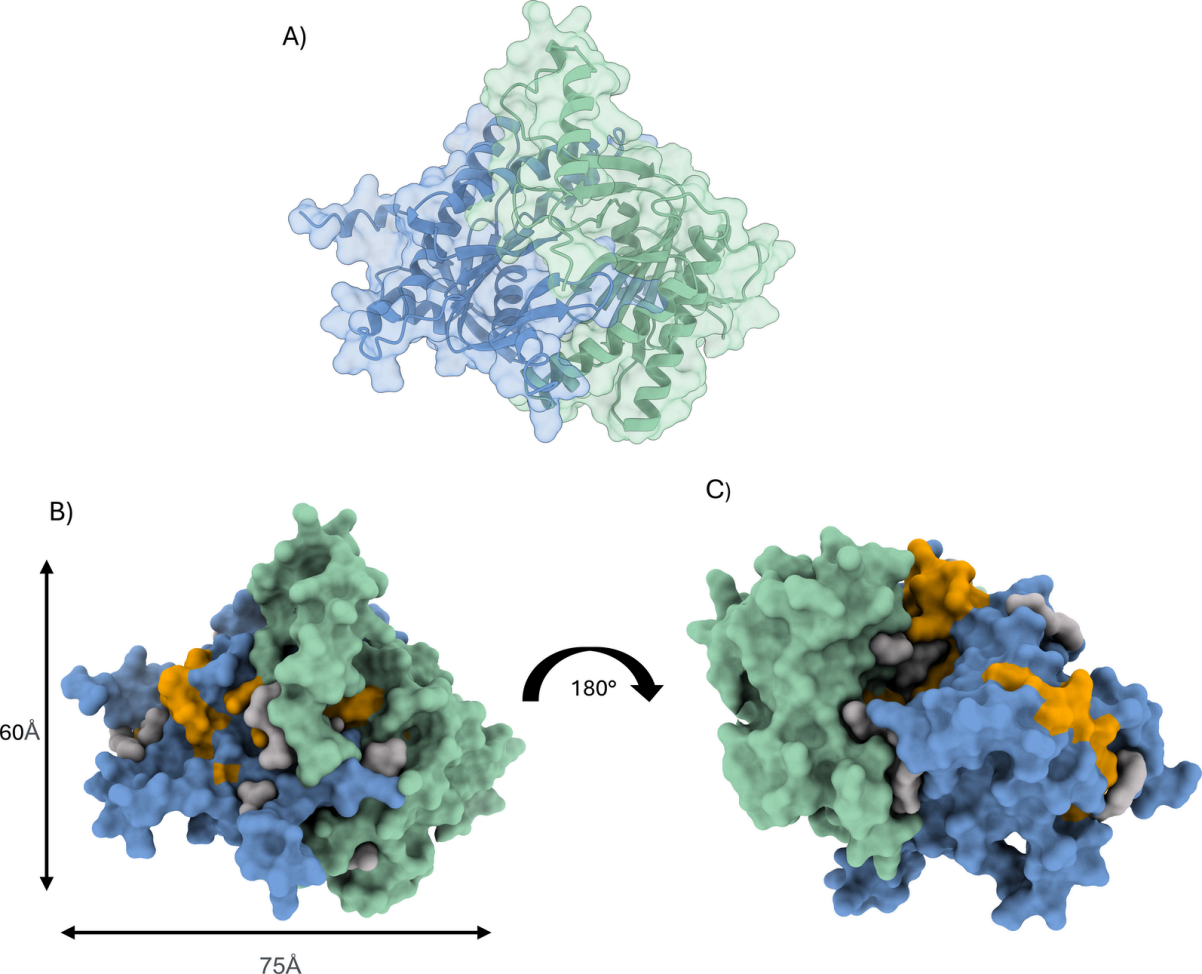
Predicted model of AC7 with cytosolic subunits C1 (green) and C2 (marine). Gray volumes indicate predicted binding sites. Identified CARC and CRAC motif residues are marked in orange color. (A) 3D model of AC7 with highlighted secondary structure elements (cartoon). (B) Surface model representation of the AC7 model showing dimensions of the model. (C) Surface model representation rotated by 180° relative to panel (B).

**Table 2:** List of Identified CARC and CRAC Motifs in the Cytosolic Subunits of ACs^a^

parameter	motif	position (AC7—*UniProtKB*)	presence
**1**	K-ILGDC-YYC-V	324	AC1–AC8
**2**	RKWQ-F/Y-DV	388	AC1–AC9
**3**	KV-FY-TECD-V	889	AC2, AC7
**4**	KPK-F-SGV	921	AC2, AC4, AC7
**5**	KTIGST-Y-MAAAGL	931	AC1–AC9
**6**	L-G-Y-SCEC-R	1044	AC2, AC4, AC7

a The position of each motif is denoted as a starting residue according to the AC7 sequence.

The first identified CARC motif, _324_K-ILGDC-YYC-V, is conserved across AC1 through AC8 and is situated in a region responsible for ATP and ion binding (C1:α3) (Figure 2A,B and Figure S2A). This highly conserved region lies within the C1 cytosolic subunit. A second CARC motif, _388_RKWQ-F/Y-DV, is conserved across all nine AC sequences. This motif is located approximately 10Å from the allosteric (C2:β3) site and 9Å from the ATP binding site (C1:β2) (Figure 2C,D and Figure S2B), making it important for the binding of a ligand to both the catalytic site and the FSK binding site. The 3D model of AC7 revealed a binding site surrounding this second motif, which facilitates interactions between the C1 and C2 monomers responsible for the formation of an active AC7 heterodimer. The third identified motif, _889_KV-FY-TECD-V, primarily conserved in AC2 and AC7, is located in the C2 subunit and combines the CRAC and CARC motif (Figure 2E,F and Figure S2C). Interestingly, in AC7, one CARC and one CRAC motif overlap within the same structural region (C2:α2). This helix is flanked by two predicted ligand binding sites (Figure 2F). MSA annotations also indicate that this region plays an important role in the dimerization of the C1 and C2 catalytic domains, suggesting potential functional significance for the cholesterol-binding motifs in structural assembly and regulation.

**Figure 2:**
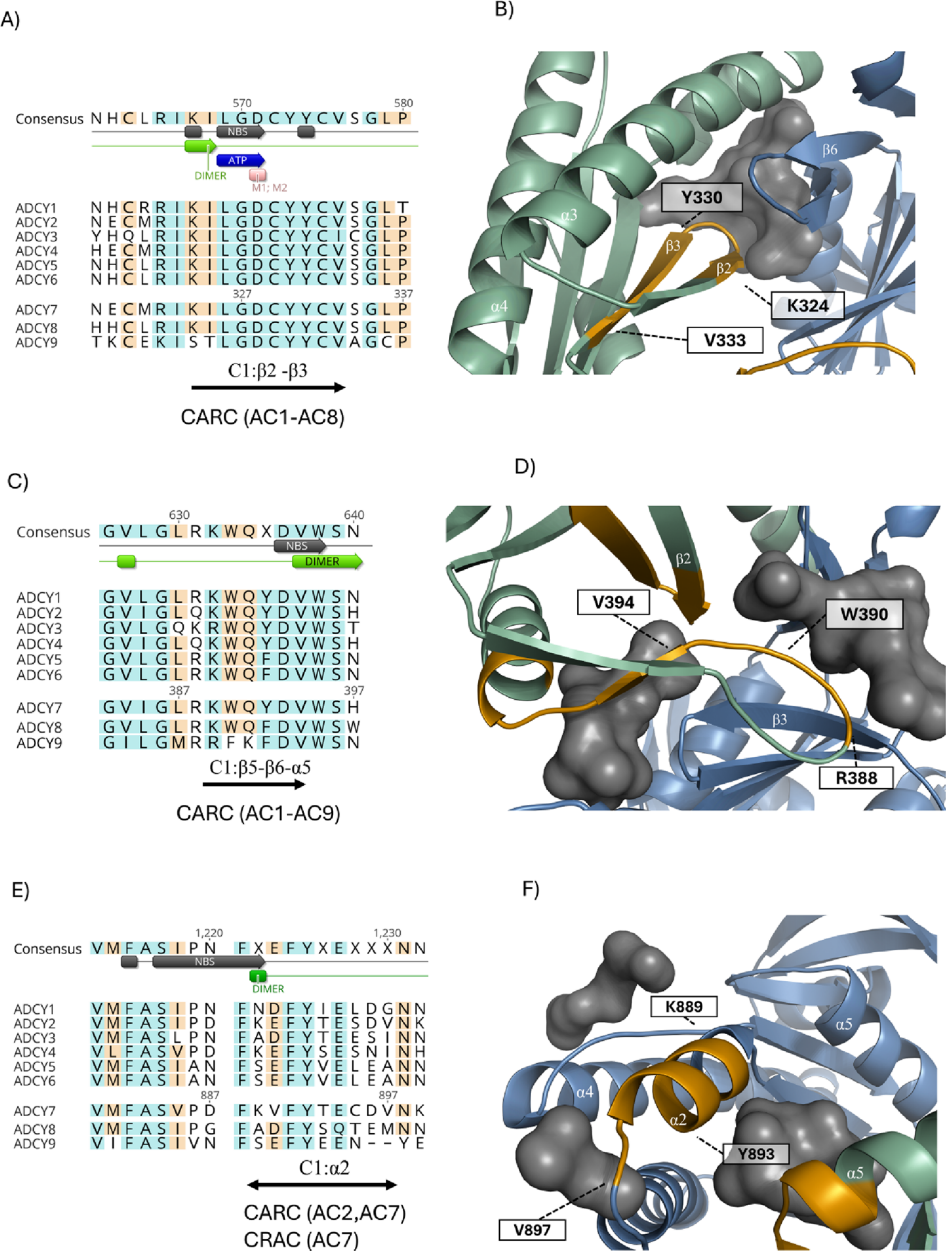
MSA of nine human isoforms of ACs, highlighting CARC and CRAC motifs. MSA colors indicate amino acid similarity, and consensus sequence shows annotated residues. The motif structural positions are highlighted in the AC7 model: C1 subunit (green), C2 subunit (marine), and predicted binding sites (gray). CARC and CRAC motifs are depicted in orange. (A) CARC motif K-ILGDC-YYC-V in a conserved region of C1, critical for ATP and Mg^2+^ binding. (B) Close-up of the K-ILGDC-YYC-V motif within the AC7 model, where the coil between β2 and β3 is responsible for ATP binding. (C) CARC motif R-KWQ-F/YD-V lies in a highly conserved region in all AC isoforms. (D) Motif R-KWQ-F/YD-V forms a coil, which supports the C1/C2 dimerization via the interaction with surrounding helices from C2. (E) Overlapping CARC and CRAC motifs; CARC is specific to AC7 and AC2, CRAC to AC7 only. (F) Both CARC and CRAC motifs lie on the same helix (α2), involved in C1/C2 dimer formation.

The fourth identified motif, _921_K-PK-F-SGV, is conserved within group 2 (consisting of AC2, AC4, and AC7) (Figure 3A,B and Figure S3A). In a very close proximity is another CARC motif, _931_K-TIGST-Y-MAAAG-L, which lies just a few residues downstream (Figure 3A,B and Figure S3A). This latter motif forms a coil between C2:β3 and C2:β4 located oppositely to CRAC on C1:β6. In short proximity to the coils lies a binding site, which is responsible for the FSK binding. Moreover, Ser935 from the second CARC motif participates in hydrogen bonding to the FSK molecule and is necessary for the proper allosteric effect of FSK. The final motif studied, _1044_L-G-Y-SCEC-R, is located at the interface of C2:α6 and C2:β10, and it is specific for AC group 2 (AC2, AC4, and AC7) (Figure 3C,D and Figure S3B).

**Figure 3:**
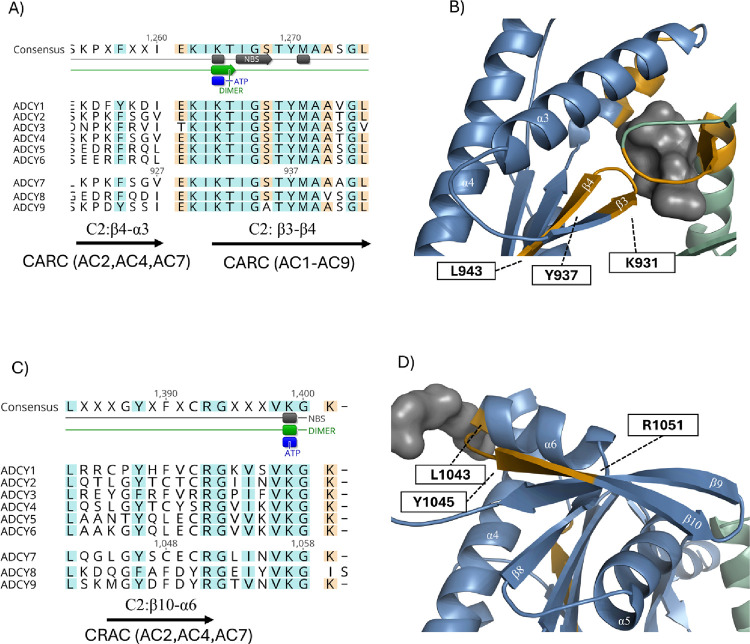
MSA of nine human isoforms of ACs, highlighting CARC and CRAC motifs. MSA colors indicate amino acid similarity, and the consensus sequence shows annotated residues. The motif structural positions are highlighted in the AC7 model: C1 subunit (green), C2 subunit (marine), and predicted binding sites (gray). CARC and CRAC motifs are depicted in orange. (A) Two CARC motifs are close: K-PK-F-SGV (specific to AC2, AC4, and AC7) and K-TIGST-Y-MAAAG-L (conserved across all ACs). (B) First CARC motif connects α3 and β3, with the second CARC motif forming a coil between β3 and β4, which is critical for FSK binding. (C) CRAC motif L-G-Y-SCEC-R, conserved in group 2 (AC2, AC4, and AC7). (D) The motif is located in non-conserved region of AC7, between α6 and β10 near a predicted binding site.

Our analysis revealed that most CARC and CRAC motifs are located in highly conserved regions of both C1 and C2 catalytic subunits. Notably, two CARC motifs in the C1 subunit are positioned near the ATP and FSK binding sites, suggesting a potential role in modulating the catalytic activity. In contrast, the motifs identified within the C2 subunit appear to be group specific. Two of the four C2 motifs are shared only among AC2, AC4, and AC7, highlighting their potential relevance for isoform-selective regulation. The presence of CARC and CRAC motifs within structurally important and functionally conserved regions reinforces their involvement in enzymatic regulation and structural stability.

### Docking of Cholesterol Revealed Multiple Binding Sites near CARC and CRAC Motifs

To explore the binding of cholesterol to AC7, we focused on five key binding sites identified through previous analysis, performing targeted docking to the predicted structure (Figure 1A–C and Figure S1). The molecule of cholesterol was docked separately at each of five identified sites near the CARC and CRAC motifs (Figures 2 and 3). The best poses of cholesterol docking were distributed across the ATP and FSK binding site. The binding energies are present in Table 3.

**Table 3:** Sum of Binding Energies for the Best Ranked Positions in Cholesterol Docking

ligand	score	T. energy	I. energy	vdW	electrostatic energy
chol_5d84	–8.506	40.226	–30.905	–21.576	–9.329
chol**_**44bc	–9.073	37.818	–33.316	–23.699	–9.617
chol_9fab	–8.759	45.963	–25.975	–19.968	–6.007
chol_00ee	–8.466	47.502	–24.279	–18.140	–6.139
ligand_94af	–7.454	45.240	–25.884	–13.856	–12.028

For example, ligand chol_5d84 was predicted to bind the enzyme’s catalytic site, interacting with two CRAC motifs located across opposing loops (Figures 1 and 4A–C and Figure S2). The cholesterol −OH group forms a hydrogen bond with the Glu328 residue, which typically binds Mg^2+^ cofactors, according to previously published structures. The highest affinity docking result was for the chol_44bc molecule, which suggested binding among C2:α3, C2:β2, and C2:α4 (Figure 4D–F and Figure S2A). Both mentioned helices directly participate in the structural changes during the AC activation either by FSK molecules or Gαs. The ligand chol_9fab tend to bind the outer leaflet between three helices (C2:α2, α3, and α4), which are part of the Gαs binding interface (Figure 5A–C and Figure S2B). The docking of chol_00ee revealed binding at the CARC motif only through hydrophobic interactions and stabilization by a small helix C2:α3 (Figure 5D–F). Lastly, chol_94af tends to bind at the interface between C1 (C1:α4) and C2 (C2:α3) subunits, forming a hydrogen bond with Asp398 (Figure 6A–C and Figure S3).

**Figure 4:**
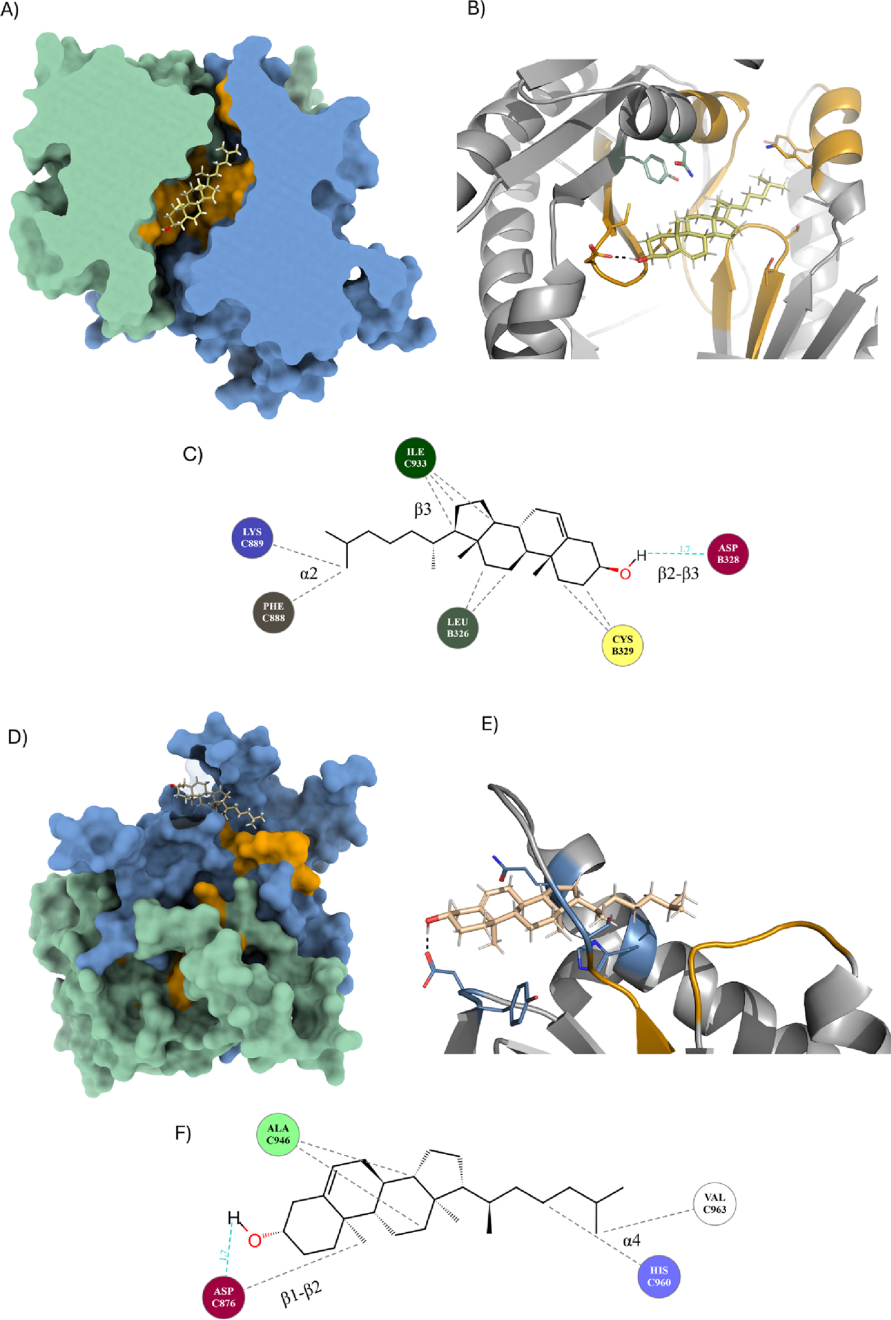
Docking of cholesterol at predicted binding sites near CARC and CRAC motifs. C1 residues are depicted as green. C2 residues are depicted as marine. (A) Surface view of AC7 with bound cholesterol (chol_5d84: yellow); CARC motifs (orange) involved in binding. Cholesterol binds preferentially in the catalytic pocket, shared by ATP and FSK binding sites. (B) Cholesterol′s binding between two coils with CARC motifs. (C) Interaction scheme showing hydrophobic interactions and a hydrogen bond with Asp328 in the K-ILGDC-YYC-V motif. (D) Surface view of the AC7 model with cholesterol (chol_44bc:brown) bound near CARC motifs. (E) Cholesterol binds in a disordered region between β3 and α3. (F) Interaction scheme showing mainly hydrophobic interactions; cholesterol’s −OH group forms a hydrogen bond with Asp876, outside of CARC or CRAC motifs.

**Figure 5:**
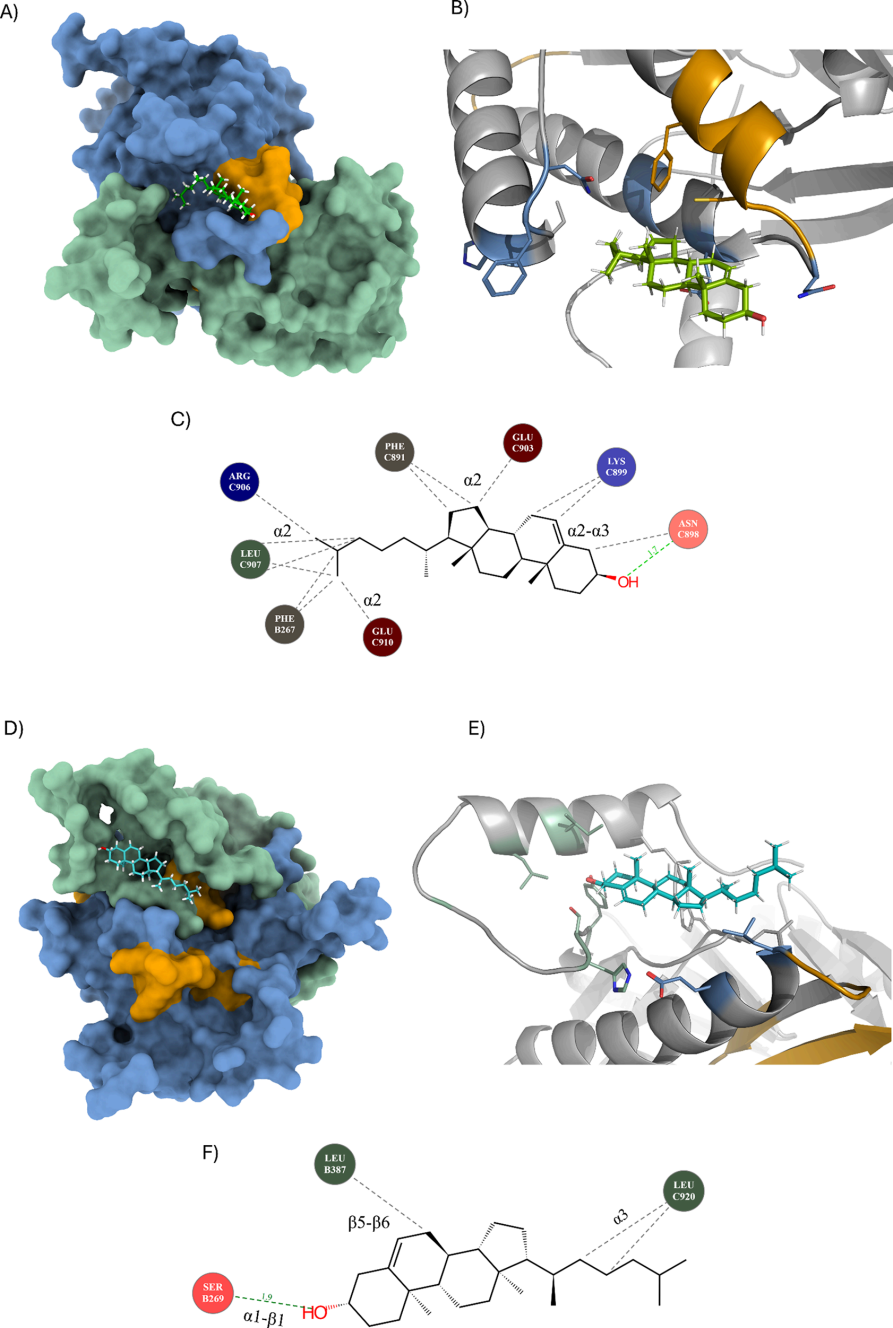
Docking of cholesterol at predicted binding sites near CARC and CRAC motifs. C1 residues are depicted as green. C2 residues are depicted as marine. (A) Surface view of AC7 with the cholesterol ligand (chol_9fab: dark green); CARC motifs (orange) involved in dimerization of C1 and C2. (B) Cholesterol’s −OH group binds to Asn898; additional binding relies on hydrophobic and stacking interactions. (C) Interaction scheme showing motif KV-FY-TECDV’s involvement in binding. Arg906 does not interact with cholesterol’s −OH group. (D) AC7 model surface view with cholesterol (chol_00ee: turquoise) bound to a flexible C1 region. (E) Cholesterol −OH group forms a hydrogen bond with Ser269, outside CARC or CRAC motifs and binds primarily through hydrophobic and stacking interactions. (F) Interaction scheme shows no participation of CARC or CRAC motif in the stabilization of cholesterol. Stabilization is dependent on the hydrophobic interactions through conserved residues on C1.

**Figure 6:**
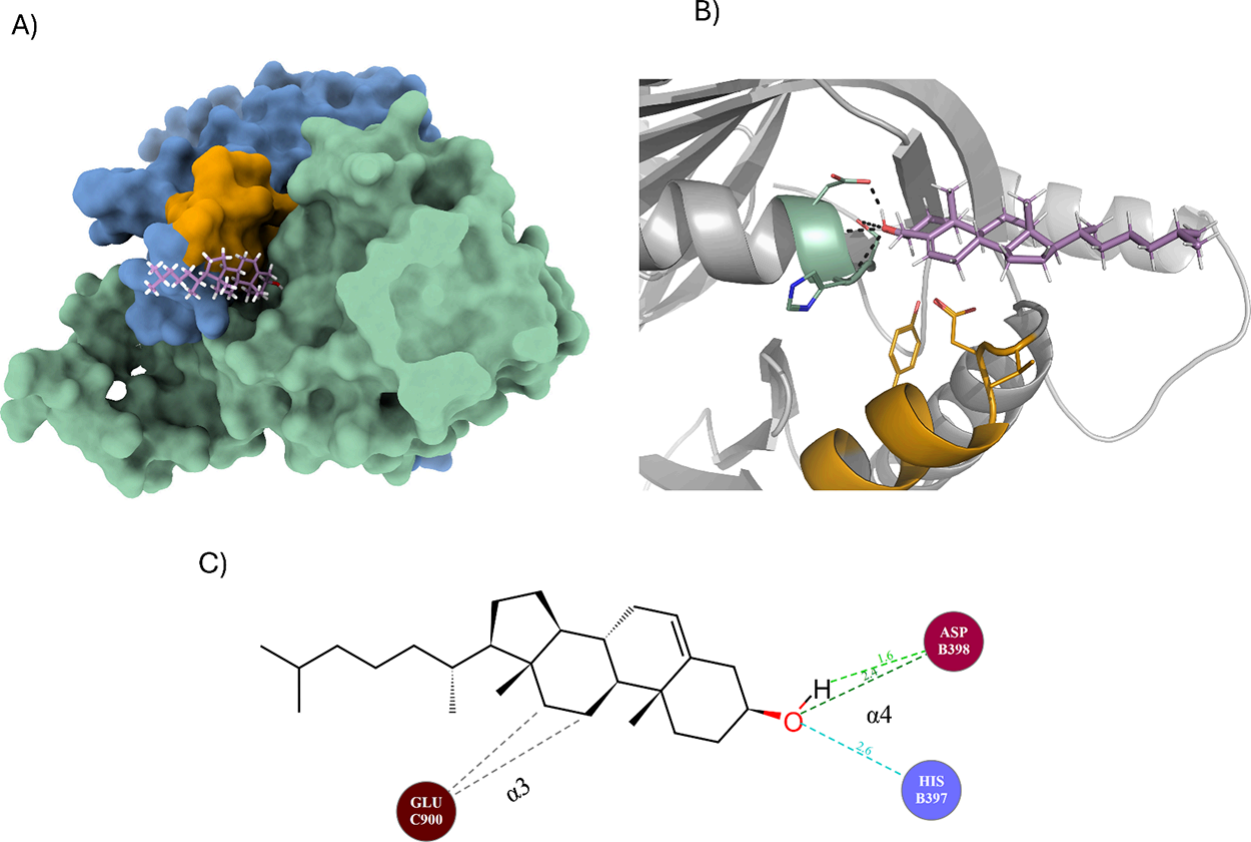
Docking of cholesterol at predicted binding sites near the CARC and CRAC motifs. C1 residues are depicted as green. C2 residues are depicted as marine. (A) Surface view of AC7 with cholesterol (ligand_94af: pink) bound to a flexible region on C2 (green). (B) Cholesterol’s −OH group forms a hydrogen bond with Asp398, outside of CARC or CRAC motifs. The molecule binding relies primarily on hydrophobic and stacking interactions. (C) Motif KV-FY-TECD-V stabilizes the aliphatic tail of cholesterol through residues from α3.

### Cholesterol Derivatives Preferentially Bind to the Allosteric Site and Catalytic Site of AC7

Given the nature of cholesterol docking, we extended our binding investigation to two *in vivo* cholesterol derivates, hydrocortisone and 25-hydroxycholesterol, and one synthetic analogue, dexamethasone (Figure 7).

**Figure 7:**
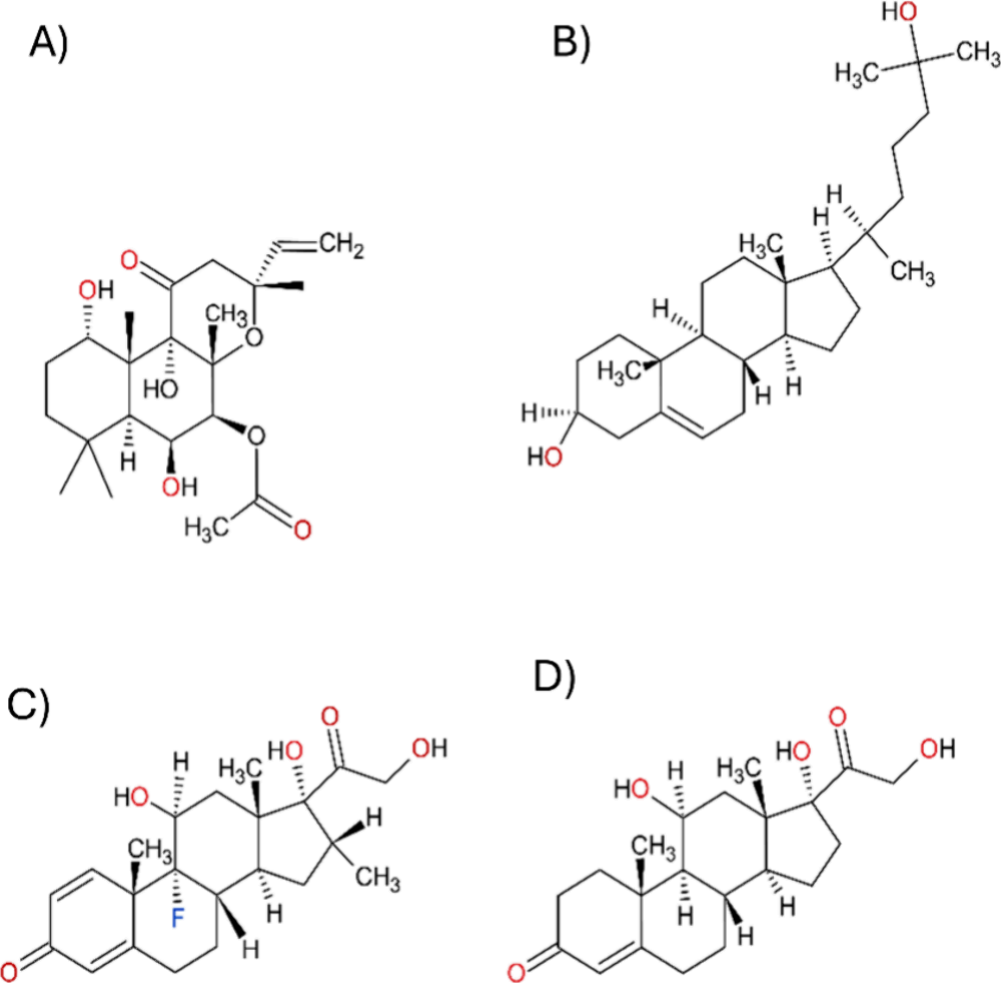
2D structures of the tested cholesterol derivatives. (A) FSK molecule used as a reference for the allosteric (FSK binding) site. FSK activates all AC isoforms nonspecifically. (B) Structure of 25-hydroxycholesterol, a native cholesterol derivative present in the brain. (C) Dexamethasone, a synthetic cholesterol derivative acting as a glucocorticoid. (D) Hydrocortisone, a natural hormone and cortisol derivative.

To reveal the binding possibilities of the chosen derivatives, we conducted a blind docking without specifying a preferable binding site. Surprisingly, the derivatives (hydrocortisone, 25-hydroxycholesterol and dexamethasone) preferentially bind to the FSK binding site (Figures 8 and 9). Moreover, all molecules utilized CARC and CRAC motif residues, which we identified within both the catalytic site and the FSK binding site of AC7 (Figures 1B, 2B, and 4B). Hydrocortisone primarily utilized the hydrogen binding with the Gly934 residue, located on the coil between C2:β3 and C2:β4, which is part of the _931_K-TIGST-Y-MAAAG-L motif (Figure 8A–C). This residue is also crucial for FSK binding and, similarly to FSK, the surrounding coil plays a key role in interaction with functional groups such as the hydroxyl group. Surprisingly, Asp393 forms a strong hydrogen bond with the second hydroxyl group. In contrast, 25-hydroxycholesterol primarily employed a hydrophobic environment. Residues such as Leu326, Phe888, and Tyr892 stabilize the molecule via stacking and hydrophobic interactions (Figure 8D–F). The only hydrogen bond is formed with Asn403 from the C1 subunit.

**Figure 8:**
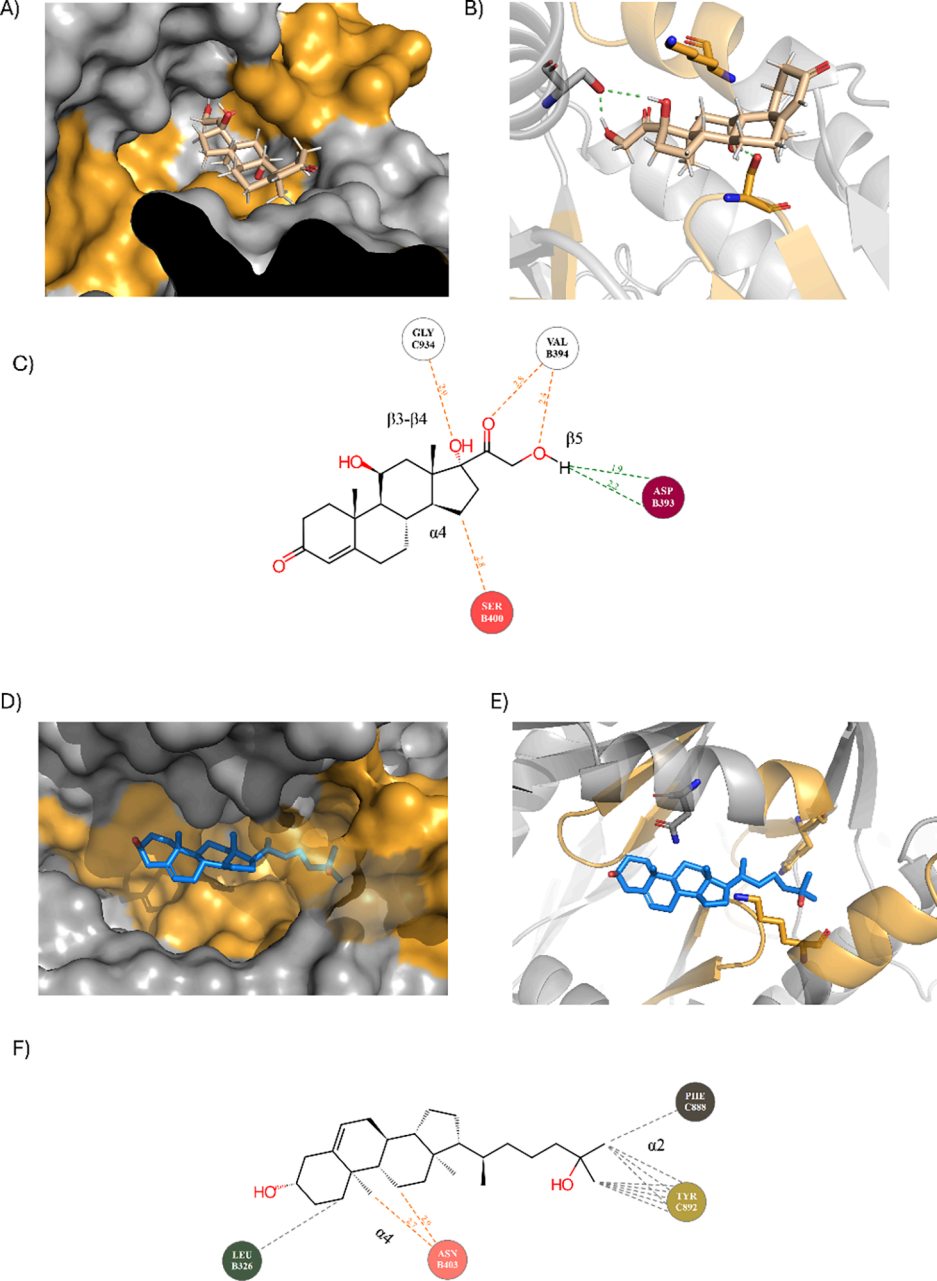
Blind docking of cholesterol derivatives hydrocortisone and 25-hydroxycholesterol, using the AC7-predicted model. CARC and CRAC motifs are shown in orange. (A) Hydrocortisone (brown) bound within the catalytic pocket combining ATP and FSK binding site. (B) Hydrocortisone primarily binds to the FSK binding site, utilizing bonds with Asp and Ser residues. The K-TIGST-Y-MAAAG-L motif forms a loop that stabilizes the molecule through hydrophobic interactions. Asp393 forms a hydrogen bond with the hydrocortisone hydroxyl group. (C) Residues Ser400 and Asp393 form the main hydrogen bonds with hydroxyl hydrocortisone groups, while aliphatic residues in the CARC motif KV-FY-TECD-V stabilize the molecule through stacking interactions. (D) 25-hydroxycholesterol (blue) prefers binding within the AC7 catalytic site across the ATP and FSK binding site. (E) 25-Hydroxycholesterol orientation is stabilized by Phe, Tyr, and Pro residues. Only a hydrogen bond was formed with Asn403, a CARC motif residue. (F) An interaction scheme shows 25-hydroxycholesterol stabilized by primarily hydrophobic and stacking interactions.

**Figure 9:**
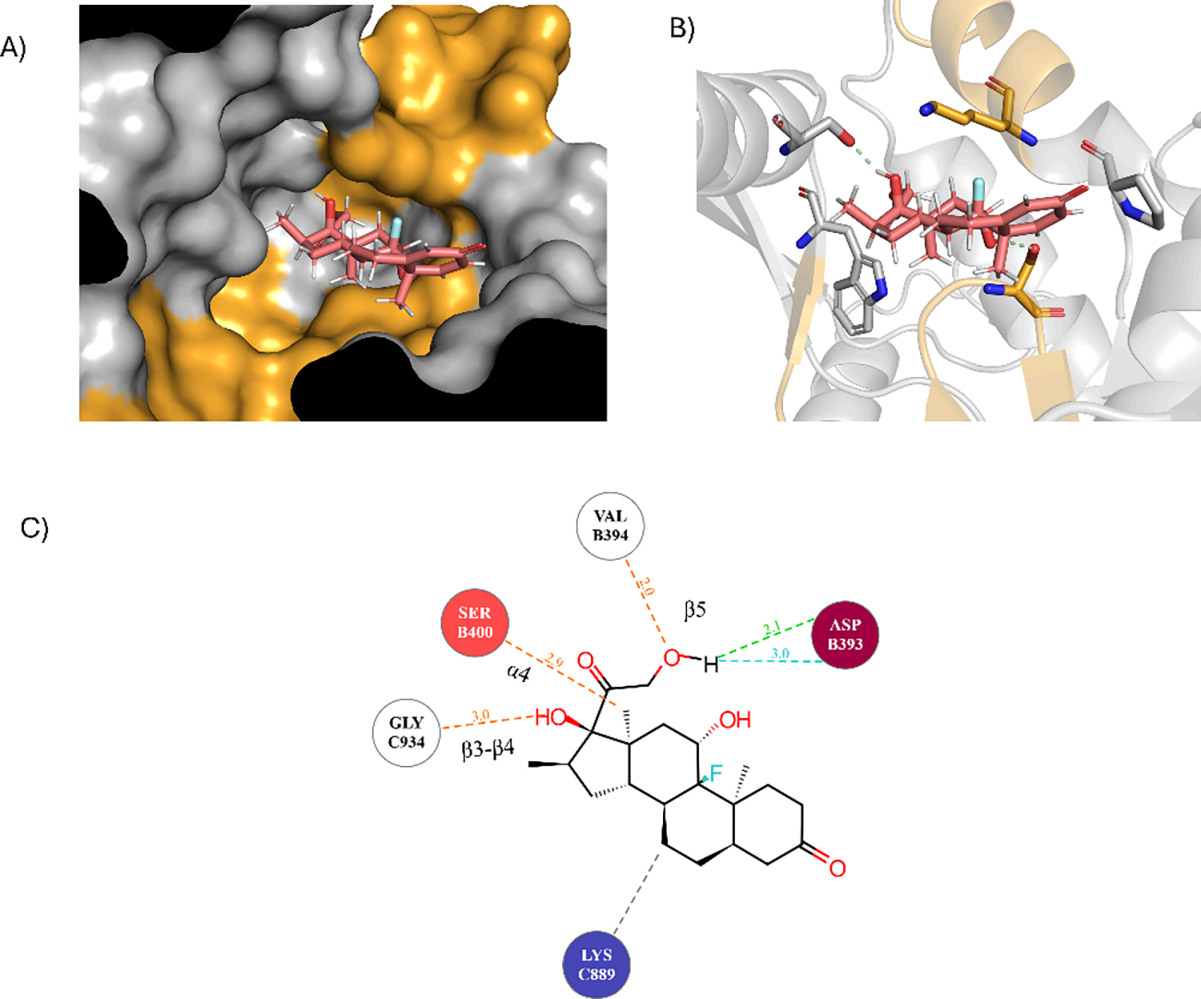
Blind docking of dexamethasone, using the AC7 model. CARC and CRAC motifs are shown in orange. (A) Dexamethasone bound within the catalytic pocket combining ATP and FSK binding site, overlapping ATP and FSK binding site. (B) Dexamethasone preferentially binds to the FSK binding site, forming bonds with charged residues Ser400 and Gly934, with hydrophobic stabilization from the K-TIGST-Y-MAAAG-L loop. (C) Dexamethasone binds strongly to the C2 subunit via hydrogen bonds. Despite its high reactivity, its fluorine atom does not interact with charged residues. Polar residues on the C1 subunit help orient functional groups toward the C2 subunit.

Dexamethasone, which also preferred binding in the FSK binding site, primarily utilized the same CARC motif as hydrocortisone. Asp393, Ser400, and Gly934 are responsible for hydrogen binding (Figure 9A–C). Notably, Ser400 is highly conserved across all nine AC isoforms (Figure 2C). Generally, the docking showed that all of these molecules preferably bind to the FSK binding site, utilizing CARC and CRAC motif residues. Binding to this site may influence AC7’s structural conformation and function, leading to an alternation of activity upon binding.

Overall, the results from docking of cholesterol derivatives highlight the preference of the binding to the FSK binding site and ATP binding site. Table 4 summarizes the best ranked positions for all ligands (derivatives).

**Table 4:** Sum of the *DiffDock* Binding Energies for the Docking of Cholesterol Derivatives

prediction	*DiffDock* confidence	smina affinity	smina intramolecular energy	smina minimized affinity	smina minimized RMSD
**rank1_hydrocotisone**	–1.33	–5.49879	–0.04163	–6.44076	0.58364
**rank1_25-hydroxycholesterol**	–1.44	–3.27954	–0.41106	–7.01311	1.22101
**rank1_dexamethasone**	–1.7	–4.98922	–0.1601	–6.6956	0.65485

### All Three Cholesterol Derivatives Significantly Decreased AC7 Activity

To experimentally validate the binding of cholesterol derivatives to AC7, we used cell membranes isolated from HEK cells with the overexpressed AC7 isoform (Figure S9A,B). Simplified membrane-based assays were chosen as the optimal experimental system. Additionally, to mitigate the effects of endogenous membrane-bound cholesterol, cholesterol was depleted in purified membranes (see Methods). The optimization of cholesterol depletion is presented in Figure S10. FSK was used as a positive control to assess the regulatory potential of the tested molecules, providing a baseline for comparison in our assays (Figure S9C). Cholesterol depletion of membranes was performed using different membrane preparations, and membranes showing the highest percentage of cholesterol depletion were selected for testing (Figure S10). Overall, cholesterol depletion resulted in only a slight decrease in AC7 activation across a range of FSK concentrations (1, 10, and 100 μM), indicating that membrane-bound cholesterol minimally contributes to FSK-stimulated activity in AC7 (Figure 10A).

**Figure 10:**
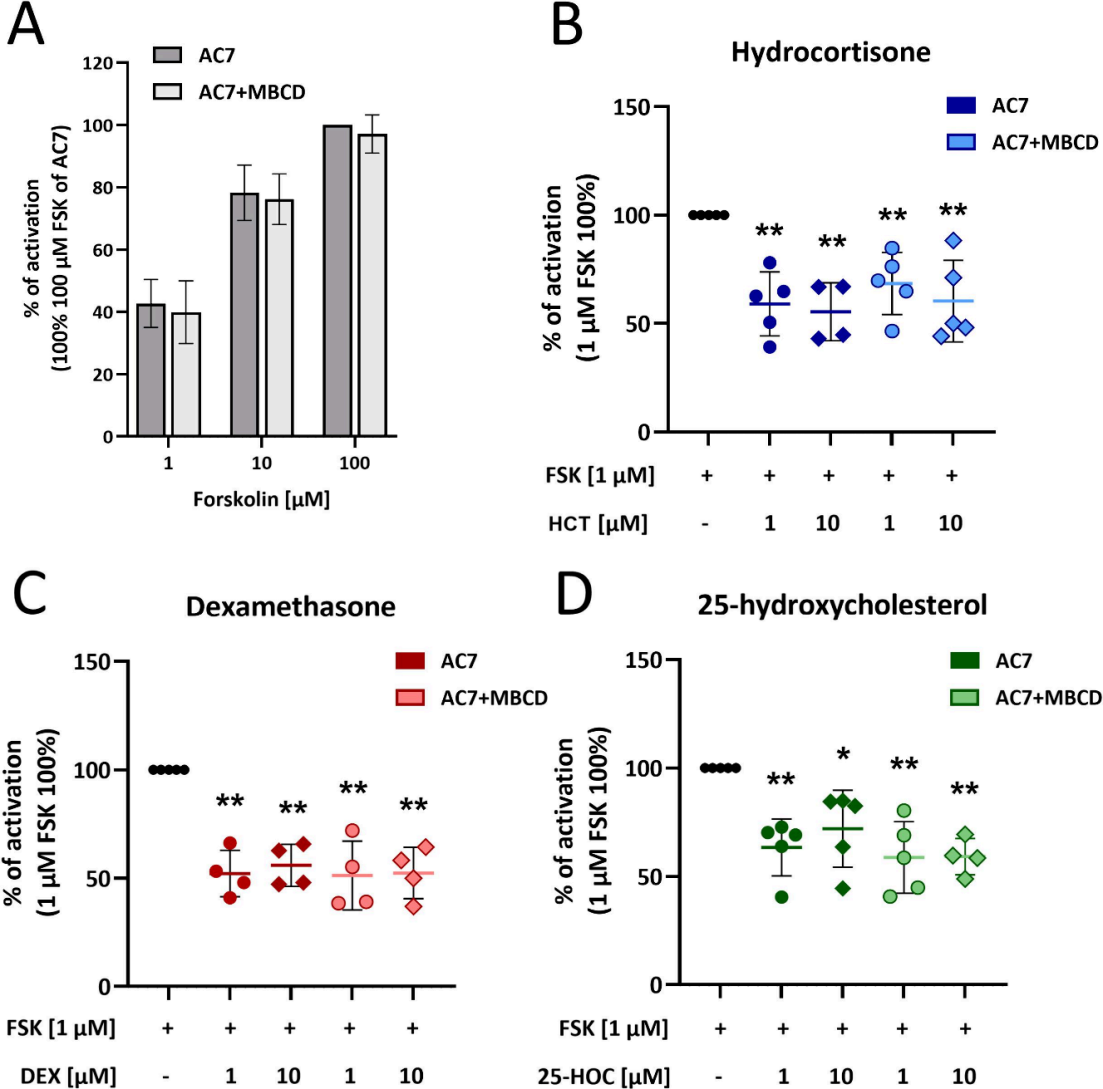
Cholesterol derivatives regulate AC7 catalytic activity by decreasing the activity upon FSK-induced activation. (A) Percentage of activation induced by FSK (1, 10, and 100 μM) of native AC7 membranes compared to AC7 membranes with depleted cholesterol (AC7+MBCD). (B–D) Evaluation of changes of FSK-induced activation (1 μM) by cholesterol derivatives hydrocortisone (HCT) (B), dexamethasone (DEX) (C), and 25-hydroxycholesterol (25-HOC) (D) (all in concentrations 1 and 10 μM) in native AC7 membranes compared to AC7 membranes with depleted cholesterol (AC7+MBCD). Statistical analysis was performed using the one-sample *t* test. Statistically significant *p*-values are reported as * *p* < 0.05, **p < 0.001, *n* = 4–5.

Experimental results demonstrated that hydrocortisone treatment led to an approximately 45% reduction in FSK-induced AC7 activity at both 1 and 10 μM hydrocortisone concentrations (Figure 10B). Both cholesterol-depleted and -nondepleted membranes were sensitive to hydrocortisone, with only minor differences observed between the two concentrations (Figure 10B). Similarly, dexamethasone exhibited an inhibitory effect, reducing the level of FSK-induced AC7 activation by 50% (Figure 10C). The 25-hydroxycholesterol treatment resulted in a decrease in AC7 activity, ranging from 25 to 45%, with the highest reduction observed at 10 μM in depleted membranes (Figure 10D). Overall, all three cholesterol derivatives tested, hydrocortisone, dexamethasone, and 25-hydroxycholesterol, significantly decreased AC7 activity induced by FSK. Notably, the extent of the inhibition did not consistently follow a concentration-dependent trend, as the most prominent decreases were often observed at the lower concentration (1 μM), particularly in cholesterol-depleted membranes (Figure 10B–D).

## Discussion

In this study, all CARC and CRAC motifs within the cytosolic domains of human AC isoforms (AC1–AC9) were identified and mapped by using an *AlphaFold2*-predicted structure of AC7. Based on the MSA and AC7 model, these motifs are located at the catalytic site, the FSK binding site, and even in the close proximity of the Gαs binding interface.

Altogether, nine CARC and CRAC motifs were identified in the cytosolic subunits of different AC isoforms. Some of the identified CARC and CRAC motifs are AC group specific, mainly in the case of the type 2 group (AC2, AC4, and AC7). Interestingly, the remaining transmembrane parts of ACs do not contain any CARC and CRAC motifs, as we can conclude on the basis of protein sequence analysis. We assume that the presence of specific motifs within the cytosolic domain of ACs should be discussed in terms of the particular group of isoforms, since each group shares primary sequence similarity and their own specific forms of regulation.^1,3,5^ When we focused on AC7, the primary sequence contains two CARC motifs in the C1 subunit, and four motifs are present in the C2 cytosolic subunit. Based on the predicted model, the localization of those motifs was within the catalytic site, the FSK binding site, and even in the Gαs binding interface. The distribution of CARC and CRAC motifs supports their role as integral structural features of AC7, contributing to the maintenance and regulation of the catalytic activity.

To reveal whether cholesterol molecules can bind to the identified sites near CARC and CRAC motifs, we used targeted docking to reveal which amino acid residues participate in the binding. Our results showed that CARC and CRAC motif residues may participate in the binding of cholesterol to the AC7 model, especially in the case of motifs located within or near the catalytic site and FSK binding site. In contrast to targeted docking, we applied blind docking to clarify the preferential binding regions. We widened our analysis to the study of sterol-type ligands that may interact with CARC and CRAC motifs. Since cholesterol is a precursor of many molecules, we decided to study three derivatives: hydrocortisone, an endogenous analogue of cortisol that regulates stress responses and metabolism; 25-hydroxycholesterol, which plays a critical role as a signaling molecule in various biological processes; and dexamethasone, which is a potent glucocorticoid commonly used in medicine for its anti-inflammatory and immunosuppressive properties.

The docking revealed a preferential binding site for all three derivatives, which was within the FSK binding site. Hydrocortisone demonstrated a primary reliance on hydrogen bonds, positioned within the coil between two conserved beta sheets, which are necessary for AC activation via the FSK molecule (Figure 8C). Moreover, dexamethasone also favored binding at the FSK binding site (Figure 9), utilizing the same residues as hydrocortisone, likely due to the high structural similarity between both molecules. The two main motifs that participated in the binding of both molecules were _931_K-TIGST-Y-MAAAG-L and _889_KV-FY-TECD-V. Residues from both motifs are known to be necessary for the proper binding of FSK.^10,14^ In contrast, 25-hydroxycholesterol revealed a preference for a hydrophobic environment, but the most favorable binding spot lies between the ATP and FSK binding sites.

The structural mapping of CARC and CRAC motifs in the AC7 model revealed their presence in regions accessible to small sterol ligands. These motifs were located within key secondary structural elements of the C1 and C2 subunits, including the C1:α2 and C1:α3 helices, both of which are crucial to the AC activation mechanism.^10,11^ The blind docking analysis in this study showed that sterol ligands engage with CARC and CRAC motifs positioned within both the allosteric (FSK binding) and catalytic (ATP binding) regions. These docking results, combined with functional assays, demonstrated that cholesterol derivatives interact with AC7 and modulate its enzymatic activity. The localization of CARC and CRAC motifs in these structurally and functionally critical regions underscores their importance in maintaining catalytic integrity and responsiveness to binding of FSK, which leads to big conformational changes.^6,35,36^

Experimental evaluation of AC7 activity demonstrated that cholesterol derivatives influence enzymatic output following forskolin (FSK) activation. After stimulating AC7 with FSK, the addition of hydrocortisone, dexamethasone, and 25-hydroxycholesterol resulted in a reduction of AC7 activity by 25–55% relative to FSK alone. Comparable levels of inhibition were observed in both cholesterol-depleted and non-depleted membranes. Membrane depletion using MBCD reduced the cholesterol content to approximately 40% of baseline levels, but AC activity was preserved, indicating the structural stability of AC7 in these conditions. Given these findings, a membrane-based assay was selected as the experimental platform, allowing for an accurate assessment of enzymatic regulation. The tested sterol derivatives consistently reduced AC7 activity, confirming their inhibitory effects in the context of FSK-induced activation. These results highlight the ability of cholesterol-related ligands to modulate AC7 function without acting as direct activators, in contrast to the strong stimulatory effect of FSK. Moreover, a negative regulation of AC7 primarily occurs through interactions with soluble proteins like calmodulin kinases (e.g., CAMK2).^37^

The conservancy and placement of these motifs are consistent with broader patterns seen across the AC family, where structural motifs often reflect adaptation to maintain precise regulation of activity.^1,3^ While the evolutionary origins of these motifs remain under investigation, their conservancy suggests an importance in supporting both structural integrity and responsiveness to cellular signals.^38–40^

## Conclusions

In conclusion, this study identified cholesterol-binding CARC and CRAC motifs in conserved cytosolic regions of AC isoforms, expanding the current understanding of their distribution beyond membrane-associated contexts. *In silico* analyses demonstrated that cholesterol derivatives hydrocortisone, dexamethasone, and 25-hydroxycholesterol may consistently interact with CARC motifs located near the FSK binding site. Functional assays using membranes overexpressing AC7 demonstrated that these derivatives reduced FSK-stimulated AC7 activity by up to 55%. These results provide evidence that cholesterol derivatives can influence AC7 function, supporting a modulatory role under the tested conditions. Together, the data reinforce the structural and functional relevance of CARC and CRAC motifs in AC7 and contribute to a deeper understanding of how sterol-related molecules may impact AC activity. Further studies are needed to clarify the exact mechanisms behind the observed inhibition and broader physiological implications of our findings.

## Limitations

The primary findings are based on *in silico* structural predictions and docking analyses, which, while informative, require experimental validation to confirm precise ligand binding interactions. Although functional assays demonstrated an inhibitory effect of cholesterol derivatives on AC7 activity, direct evidence of binding at specific CARC and CRAC motifs remains to be established. Future work involving the site-directed mutagenesis of these motifs will be essential to determine their exact role in ligand binding and AC7 regulation. Additionally, the current experiments were limited to overexpression systems in membrane preparations, and further investigations in physiological and *in vivo* contexts are necessary to fully assess the biological significance of these findings.

## Supplementary Material



## Data Availability

All data from *in silico* analyses are available on 10.5281/zenodo.14540567 or GitHub repository https://github.com/RadJarous/AC7_regulation.

## Notes

Amino acid sequence used for AC7 modeling, secondary structure elements of AC7, predicted 3D model of AC7 cytosolic domains, visualization of CARC and CRAC motifs, docking simulations of cholesterol at predicted binding sites, interaction schemes illustrating motif involvement, AC7 clone validation by gene and protein expression, forskolin derivative activity comparisons, and cholesterol depletion optimization and quantification in AC7 membranes (PDF)

## #

Fondazione Istituto Nazionale di Genetica Molecolare, Romeo ed Enrica Invernizzi, Via Francesco Sforza 35, 20122 Milan, Italy (P.D.)

## ‡

Federal University of Rio de Janeiro, Avenida Pedro Calmon 550, Cidade Universitária, 21941901, Rio de Janeiro, Brazil (L.D.).
