# Supplementary material for: Cholesterol Derivatives Regulate Adenylyl Cyclase 7 Activity by Binding CARC and CRAC Motifs in the Cytosolic Subunits
Source: ACS Omega. 2025 Aug 14;10(33):37039–52. doi: 10.1021/acsomega.5c00741 (PMC12391971; doi:10.1021/acsomega.5c00741)
Supplement: Supplementary file 1 [file ao5c00741_si_001.pdf]

Cholesterol derivatives regulate Adenylyl cyclase 7 activity by binding CARC and CRAC motifs in the cytosolic subunits

Radim Jaroušek[1,2], Petra Daďová [1,2#], Alexandra Litvinchuk[2], Leticia Dobler[2‡], Lukáš Kubala[1,2,3*]

[1]*Masaryk University, Department of Experimental Biology, Faculty of Science, Kotlářská 2,*

602 00, Brno, Czech Republic.

[2]*Institute of Biophysics, Czech Academy of Sciences, Department of Biophysics of the Immune System, Královopolská 135, 612 00, Brno, Czech Republic*

[3]*St. Anne's University Hospital Brno, International Clinical Research Center, Center of Biomolecular and Cellular Engineering, Pekařská 53, 656 91, Brno, Czech Republic*

*Email: kubalal@ibp.cz; Phone: +420 541 517 117

Table S1: *Amino acid sequence used for the 3D model of AC7 heterodimer(C1:C2).*

C1:AlphaFold2	*C2:AlphaFold2*
MPAKGHISMGMKLAIIERLKEHGDRRCMPDNNF<br>HSLYVKRHQNVSILYADIVGFTQLASDCSPKELVVV<br>LNELFGKFDQIAKANECMRIKILGDCYYCVSGLPVS<br>LPTHARNCVKMGLDMCQAIKQVREATGVDINMR<br>VGIHSGNVLCGVIGLRKWQYDVWSHDVSLANRM<br>EAAGVPGRVHITEATLKHLDKAYEVEDGHGQQRD<br>PYLKEMNIRTYLVID	GDKLNEDWYHQSYDCVCVMFASVPDFKVFYTEC<br>DVNKEGLECLRLLNEIIADFDELLLKPKFSGVEKIKTI<br>GSTYMAAAGLSVASGHENQELERQHAHIGVMVE<br>FSIALMSKLDGINRHSFNSFRLRVGINHGPVIAGVI<br>GARKPQYDIWGNTVNVASRMESTGELGKIQVTEE<br>TCTILQGLGYSCECRGLINVKGKGELRTYFVCTDTA<br>KFQGLGLN

Table S2: *Secondary structure elements for both subunits of AC7 (C1;C2). The secondary structure regions are based on predicted model.*

**C1**		**C2**	
243–256	α1	864–871	α1
272–286	β1	872–875	β1
288–294	α2	878–884	β2
297–318	α3	885–895	α2
321–326	β2	902–920	α3
329–334	β3	928–933	β3
343–365	α4	936–941	β4
370–378	β4	954–981	α4
379–383	β5	989–995	β5
393–395	β6	997–1002	β6
397–408	α5	1008–1012	β7
413–417	β7	1014–1025	α5
418–424	α6	1030–1034	β8
435–445	α7	1035–1044	α6
		1048–1056	β9
		1059–1067	β10

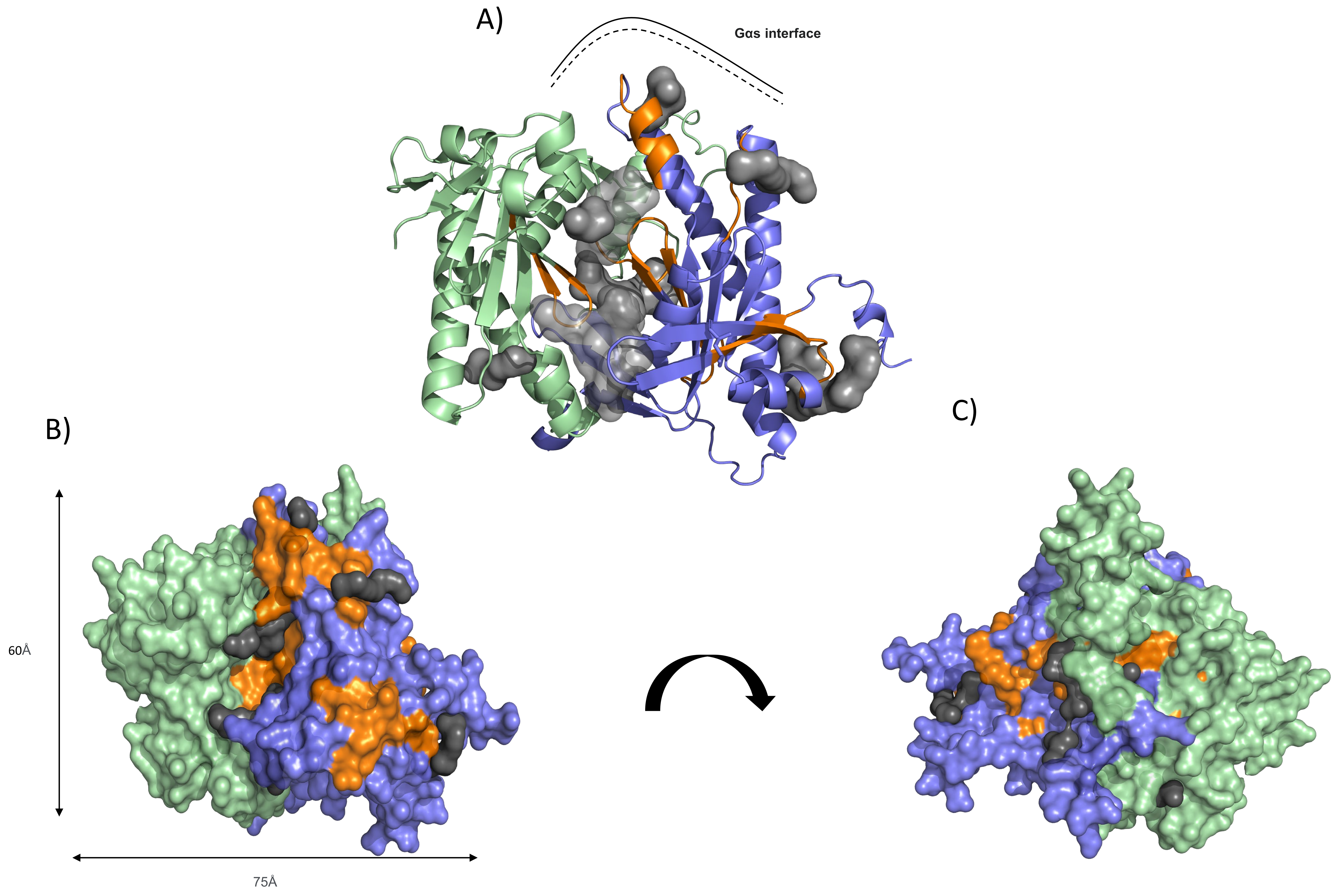

***Figure S1**: Predicted model of AC7 cytosolic subunits C1 (green) and C2 (marine). Gray volumes indicate predicted binding sites. CARC and CRAC motif residues are marked in orange color.*

***A)** AC7 model with highlighted secondary structure elements.*
***B)** A surface representation of AC7 model*
C)** A surface representation of AC7 model 180° relative to **B)

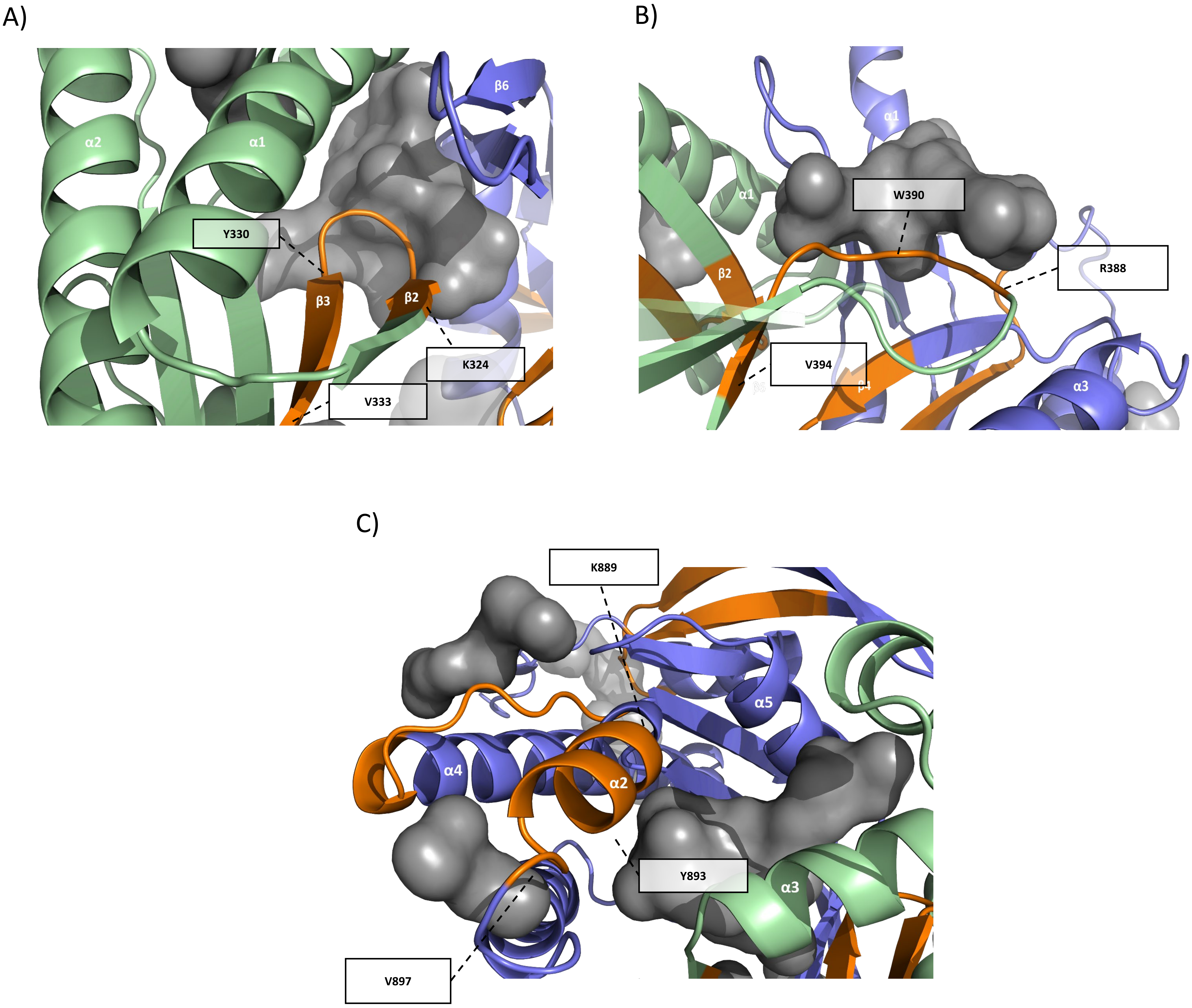

Figure S2: *Visualization of CARC and CRAC motif positions in the AC7 model. The C1 subunit is depicted as green. The C2 subunit is depicted as marine, and predicted binding sites as gray. CARC and CRAC motifs are depicted in orange.*

A) *Close-up of the K-ILGDC-YYC-V motif within the AC7 model, where the coil between β2 and β3 is responsible for ATP binding.*
B) *Motif R-KWQ-F/YD-V forms a coil which supports the C1/C2 dimerization via interaction with surrounding helices from C2.*
C) *Both CARC and CRAC motifs lie on the same helix (α2), involved in C1/C2 dimer formation.*

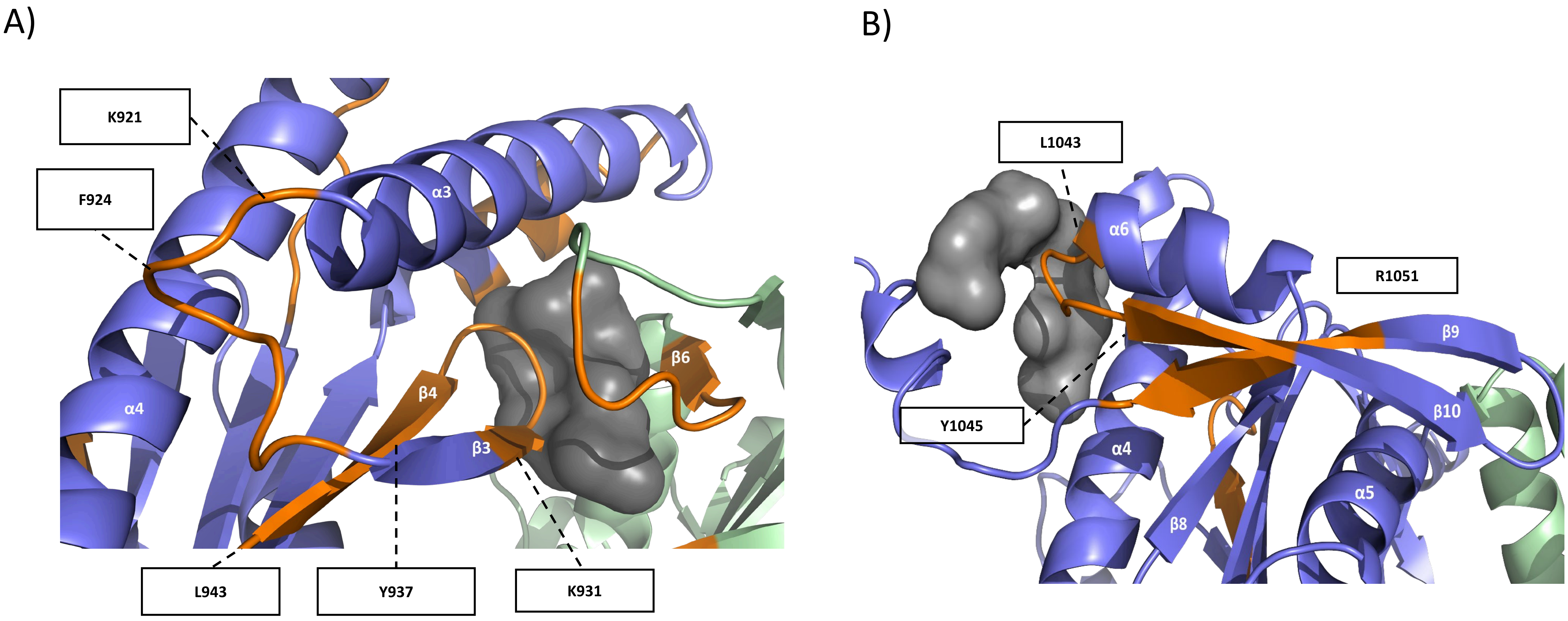

Figure S3: *Visualization of CARC and CRAC motif positions in the AC7 model. The C1 subunit is depicted as green. The C2 subunit is depicted as marine, and predicted binding sites as gray. CARC and CRAC motifs are depicted in orange.*

A) *Two CARC motifs are close: K-PK-F-SGV (specific to AC2, AC4, and AC7) and K-TIGST-Y-MAAAG-L (conserved across all ACs). The first CARC motif connects α3 and β3, with the second CARC motif forming coil between β3 and β4 which is critical for FSK binding.*
B) *CRAC motif L-G-Y-SCEC-R, conserved in group 2 (AC2, AC4, AC7). The motif is located in non-conserved region of AC7, between α6 and β10 near to a predicted binding site.*

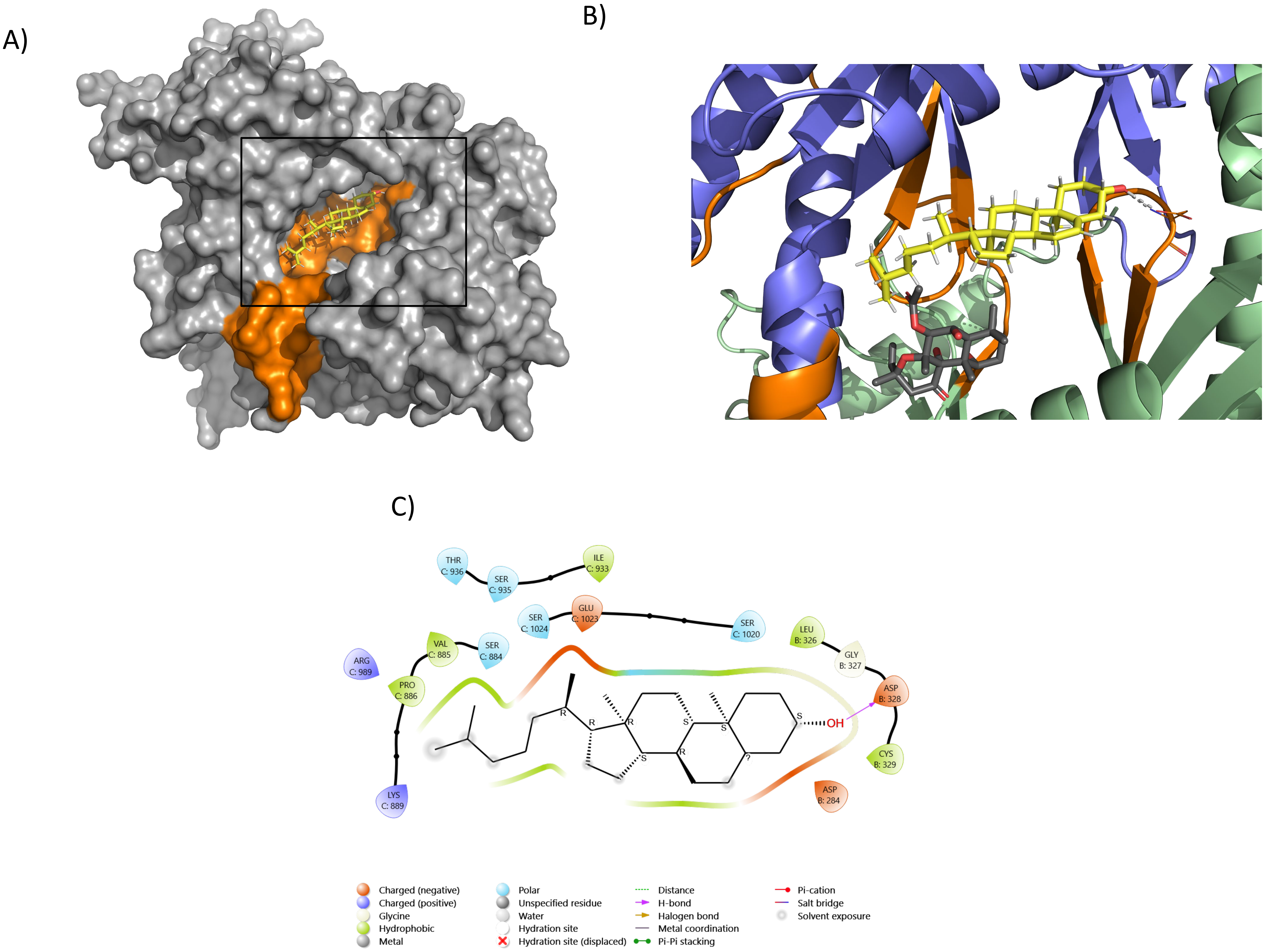

Figure S4: *Docking of cholesterol at predicted binding sites near CARC and CRAC motifs. C1 residues are depicted as green. C2 residues are depicted as marine.*

A) *Surface view of AC7 with bound cholesterol (yellow); CARC motifs (orange) involved in binding. Cholesterol binds preferentially in the catalytic pocket, shared by ATP and FSK binding sites.*
B) *Cholesterol binding between two coils with CARC motifs.*

C) *Interaction scheme showing hydrophobic interactions and a hydrogen bond with Asp328 in the K-ILGDC-YYC-V motif.*

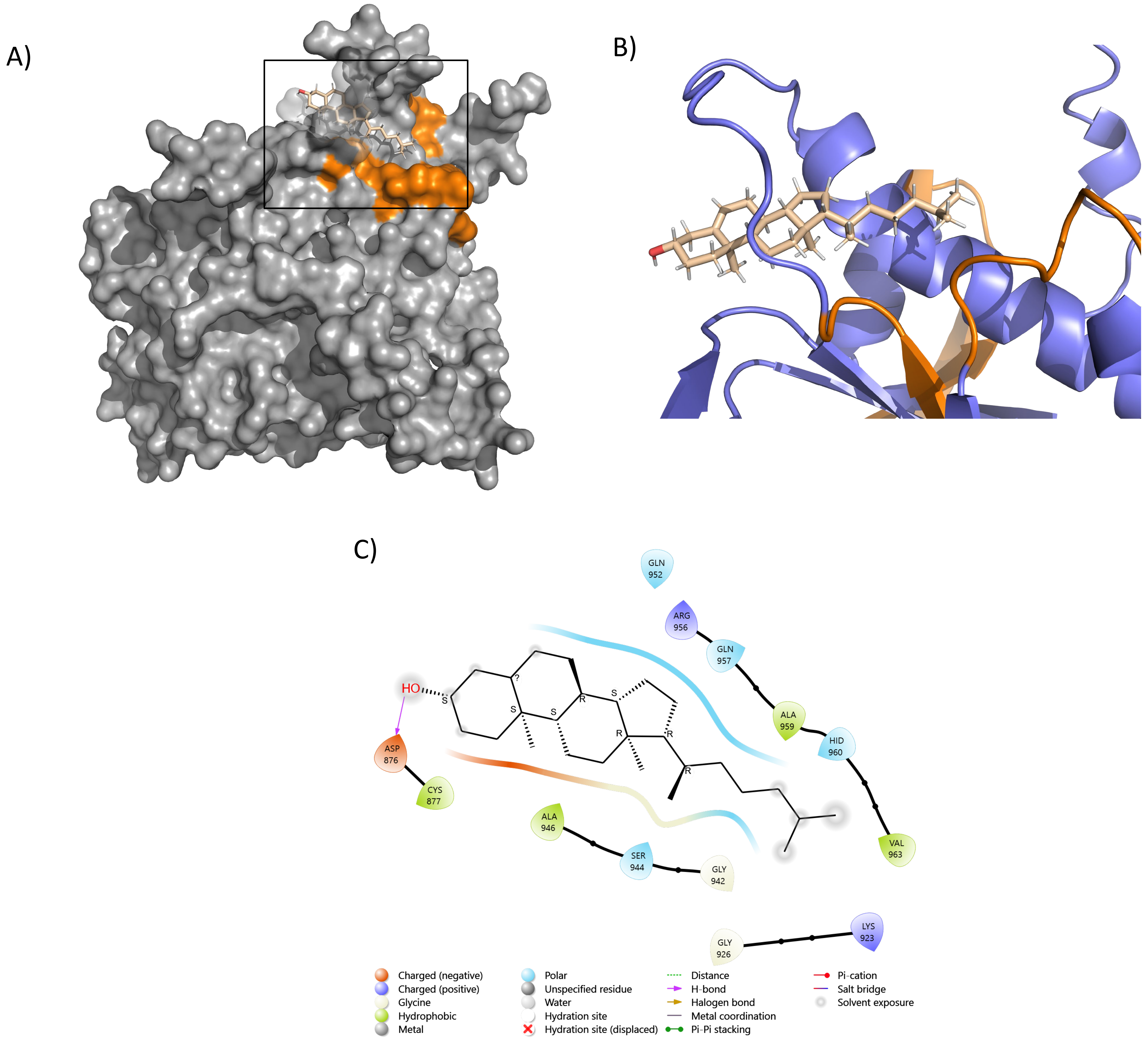

Figure S5. *Docking of cholesterol at predicted binding sites near CARC and CRAC motifs. C1 residues are depicted as green. C2 residues are depicted as marine.*

A) *Surface view of AC7 with cholesterol (brown) bound near CARC motifs.*
B) *Cholesterol binds in a disordered region between β3 and α3.*
C) *Interaction scheme showing mainly hydrophobic interactions; cholesterol's -OH group forms a hydrogen bond with Asp876, outside of CARC or CRAC motifs.*

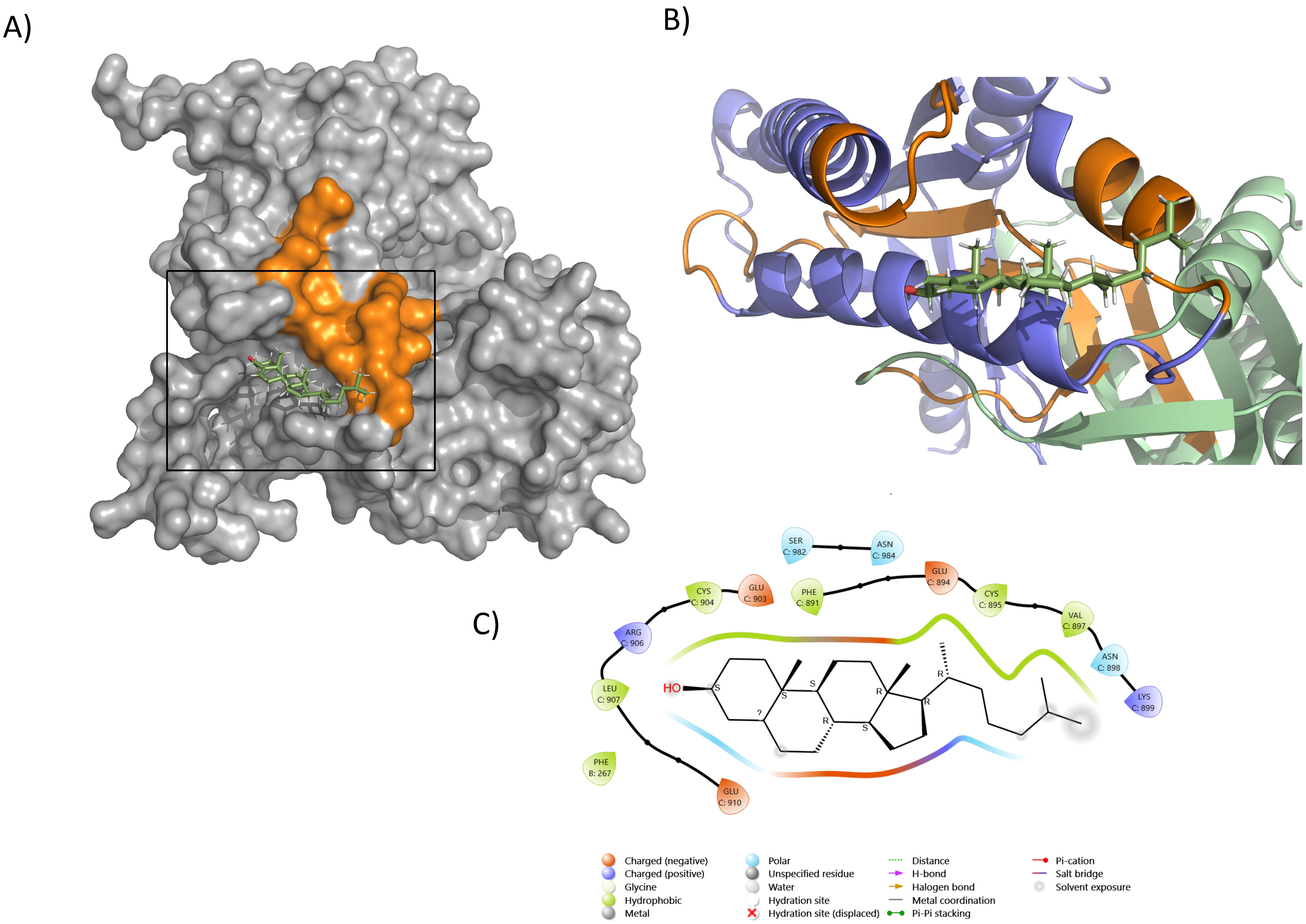

Figure S6: *Docking of cholesterol at predicted binding sites near CARC and CRAC motifs. C1 residues are depicted as green. C2 residues are depicted as marine.*

A) *Surface view of AC7 with cholesterol ligand (green); CARC motifs (orange) involved in dimerization of C1 and C2.*

B) *The binding of cholesterol relies on mainly on hydrophobic and stacking interactions.*

C) *Interaction scheme showing motif KV-FY-TECDV's involvement in binding. Arg906 does not interact with cholesterol's -OH group.*

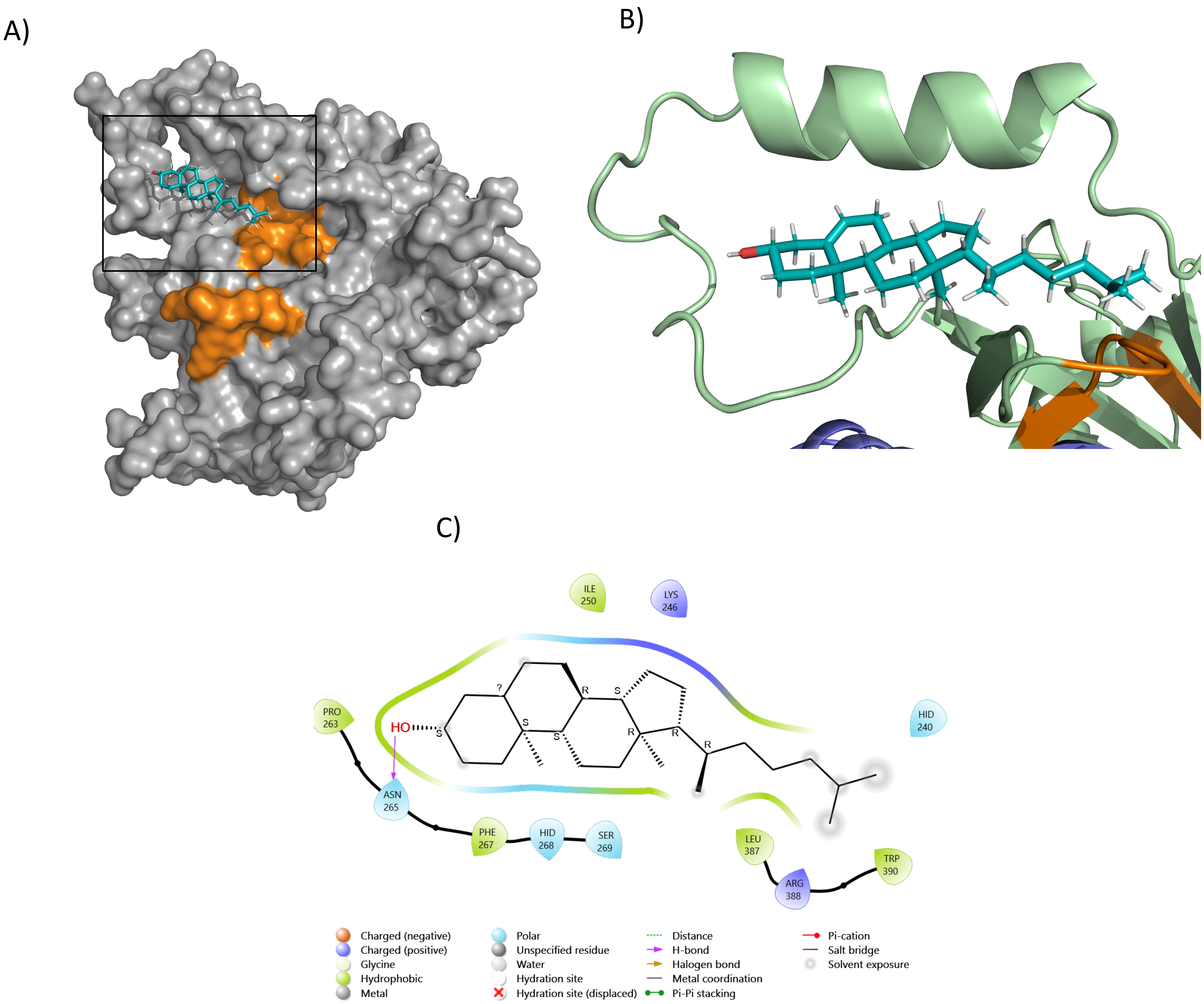

Figure S7: *Docking of cholesterol at predicted binding sites near CARC and CRAC motifs. C1 residues are depicted as darkgreen. C2 residues are depicted as marine.*

A) *AC7 model surface view with cholesterol (magenta) bound to a flexible C1 region.*
B) *Cholesterol -OH group forms a hydrogen bond with Ser265, outside CARC or CRAC motifs and binds primarily through hydrophobic and stacking interactions.*
C) *Interaction scheme shows no participation of CARC or CRAC motif in the stabilization of cholesterol. The stabilisation is dependent on the hydrophobic interaction through conserved residues on C1.*

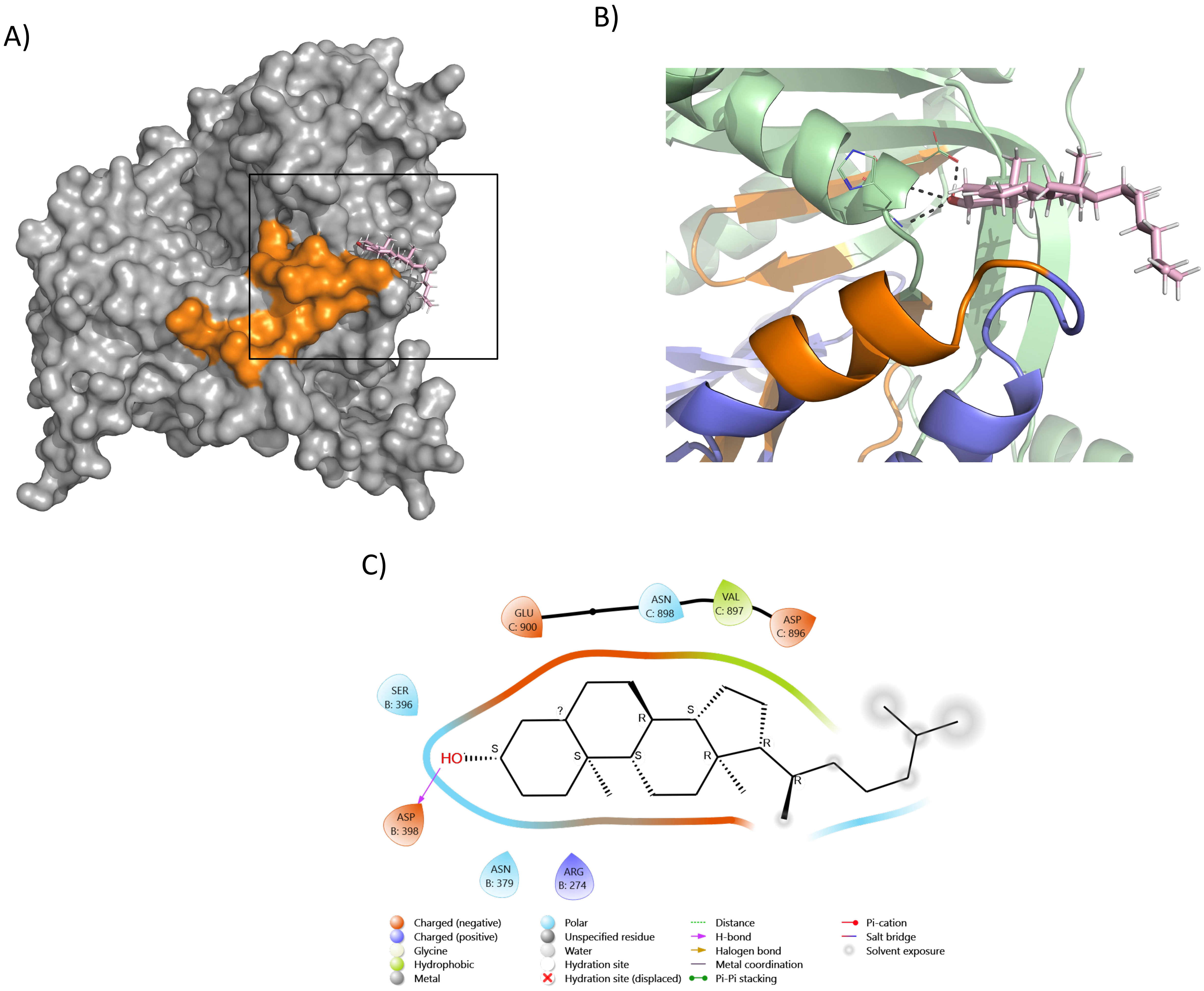

Figure S8: *Docking of cholesterol at predicted binding sites near CARC and CRAC motifs. C1 residues are depicted as green. C2 residues are depicted as marine.*

A) *Surface view of AC7 with cholesterol (pink) bound in a flexible region on C2 (darkgreen).*
B) *Cholesterol's -OH group forms a hydrogen bond with Asp398, outside of CARC or CRAC motifs. The molecule binding relies primarily on hydrophobic and stacking interactions.*
C) *Motif KV-FY-TECD-V stabilizes the aliphatic tail of cholesterol through residues from α3.*

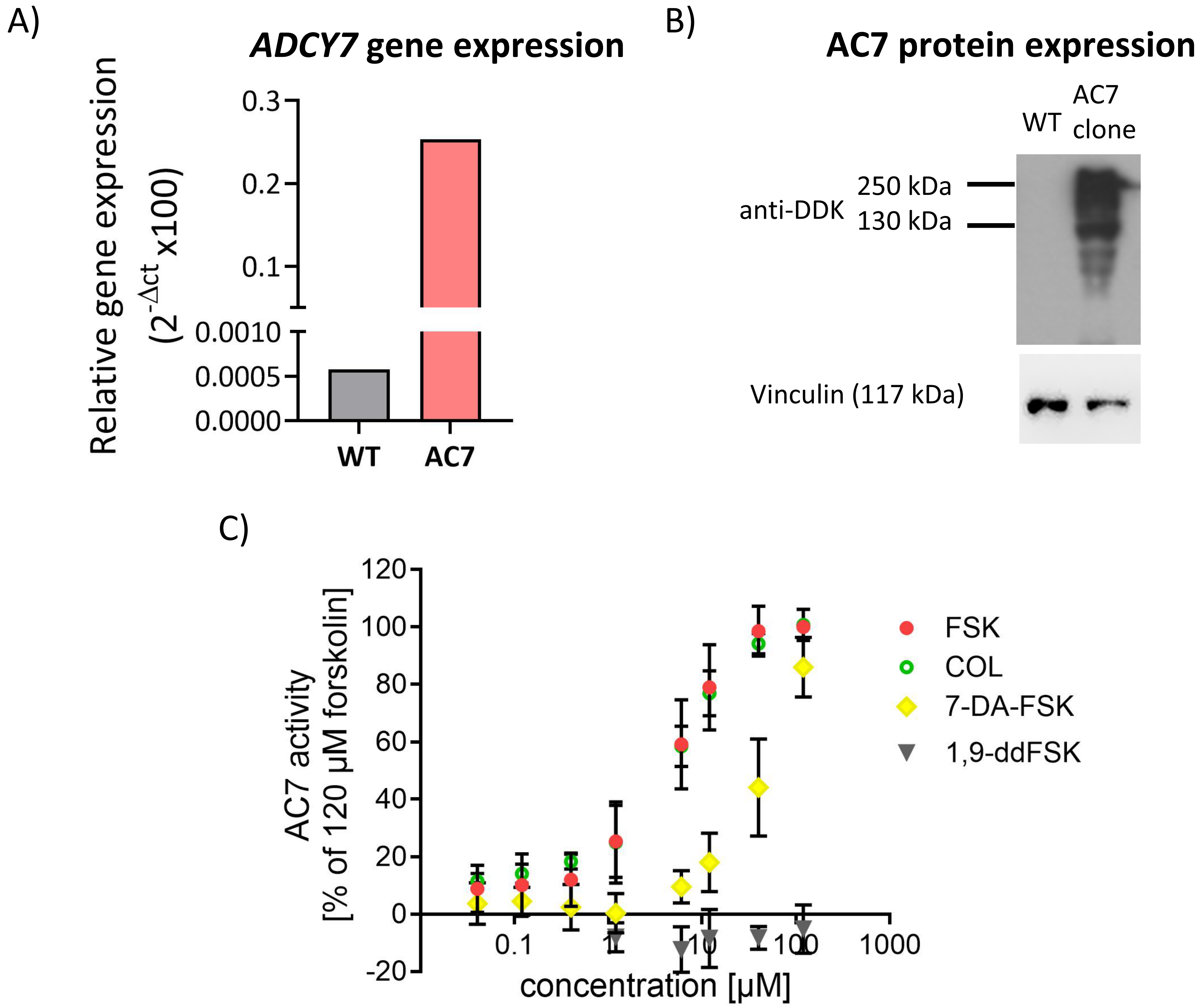

Figure S9: Verification of successful preparation of AC7 clone.

A) Determination of the relative gene expression of *ADCY7* in AC7 clone compared to WT. **B)** Representative image of western blot of the DDK-tag protein (100-250 kDa), and the reference protein vinculin (117 kDa) in control WT HEK293 cell and AC7 clone. **C)** Comparison of the effectivity of FSK and its derivatives colforsin daropate hydrochlorid (COL), 7-deacetylforskolin (7-DA-FSK), and 1,9-dideoxyforskolin (1,9-ddFSK) to stimulate AC7 activity. Saturation curves of the relative stimulatory effects of FSK and FSK derivatives (0.04-120 µM) are shown. The results were determined by dividing the stimulation obtained for distinct concen-trations of the derivate by the maximum stimulation obtained by treatment with 120 µM FSK (100%). The data are expressed as percentage (mean ± SD) of at least three independent experiments (n=3-10).

A)

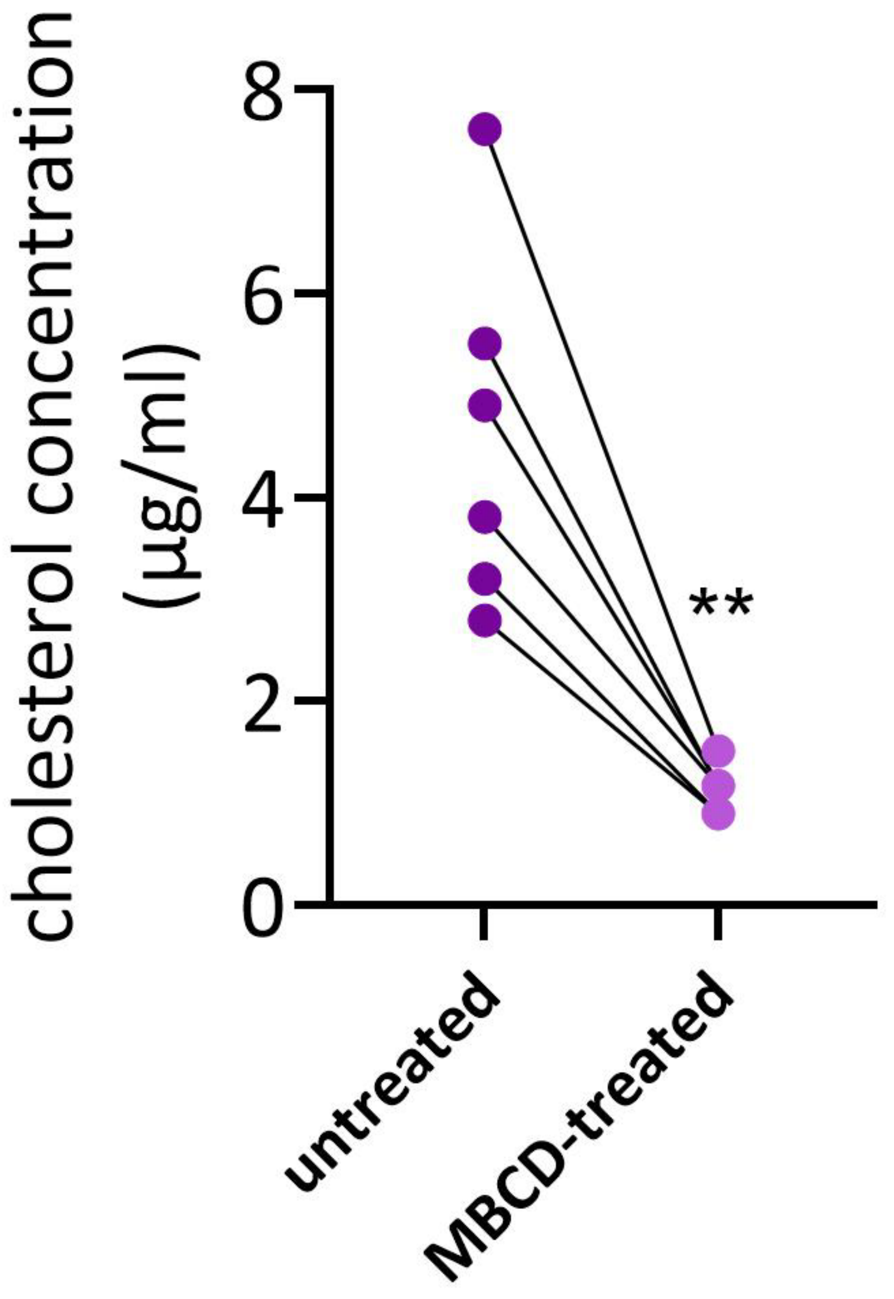

B)

No. of repetition	% of depletion
1	80.1
2	69.1
3	78.8
4	75.2
5	71.5
6	69.0
**Mean ± SD**	**74.0 ± 4.4**

Figure S10: Optimization of cholesterol depletion and detection of cholesterol concentrations in the AC7 membranes.

A) Determination of the cholesterol concentration in AC7 membranes showing the comparison between MBCD-treated and untreated (control) membranes. using *Amplex Red Cholesterol Assay Kit*.
B) Table of percentage of cholesterol depletion in AC7 membranes.
